# Hydroxyurea and inactivation of checkpoint kinase *MEC1* inhibit transcription termination and pre-mRNA cleavage at polyadenylation sites in budding yeast

**DOI:** 10.1038/s41598-023-40294-3

**Published:** 2023-08-11

**Authors:** Pritpal Kaur, Shreya Nagar, Riddhi Mehta, Kyle Sahadeo, Ales Vancura

**Affiliations:** Department of Biological Sciences, St. John’s University, 8000 Utopia Parkway, Queens, NY 11439 USA

**Keywords:** Biochemistry, Genetics, Molecular biology

## Abstract

The DNA damage response (DDR) is an evolutionarily conserved process essential for cell survival. The transcription changes triggered by DDR depend on the nature of DNA damage, activation of checkpoint kinases, and the stage of cell cycle. The transcription changes can be localized and affect only damaged DNA, but they can be also global and affect genes that are not damaged. While the purpose of localized transcription inhibition is to avoid transcription of damaged genes and make DNA accessible for repair, the purpose and mechanisms of global transcription inhibition of undamaged genes are less well understood. We show here that a brief cell treatment with hydroxyurea (HU) globally inhibits RNA synthesis and transcription by RNA polymerase I, II, and III (RNAPI, RNAPII, and RNAPIII). HU reduces efficiency of transcription termination and inhibits pre-mRNA cleavage at the polyadenylation (pA) sites, destabilizes mRNAs, and shortens poly(A) tails of mRNAs, indicating defects in pre-mRNA 3′ end processing. Inactivation of the checkpoint kinase Mec1p downregulates the efficiency of transcription termination and reduces the efficiency of pre-mRNAs clevage at the pA sites, suggesting the involvement of DNA damage checkpoint in transcription termination and pre-mRNA 3′ end processing.

## Introduction

Both exogenous and endogenous factors can generate genotoxic stress and damage cellular DNA^1–3^. Because maintenance of genome stability is crucial for survival, cells have evolved a set of highly conserved mechanisms to sense and signal damaged DNA; these mechanisms are collectively referred to as the DNA damage response (DDR)^3–5^. A major part of DDR is coordinated by the DNA damage checkpoint (DDC)^2^. In addition to DDC, eukaryotic cells have a DNA replication checkpoint (DRC) that is distinct from the DDC and signals specifically slowly progressing or arrested replication forks^2,6,7^. DDC/DRC trigger stalling or arrest of the cell cycle, initiation of DNA repair, and altered regulation of transcription, translation, and the ubiquitin–proteasome system.

The transcriptional changes elicited by genotoxic or replication stress involve RNA polymerase I, II, and III (RNAPI, RNAPII, RNAPIII)^8–16^ and depend on the nature of DNA damage, stage of the cell cycle, and DDC/DRC activation. Double-stranded DNA (dsDNA) breaks induce mostly localized inhibition of transcription around the dsDNA breaks^17^. Base damage appears to cause temporary pausing of elongating RNAPII^18^. In contrast, bulky DNA lesions caused by chemical modifications or UV irradiation elicit RNAPII stalling or arrest, leading to transcription-coupled nucleotide excision repair (TC-NER). If TC-NER is unsuccessful, Rpb1p, the largest subunit of RNAPII, is ubiquitinated and degraded as a “mechanism of last resort”^19^. In addition, DRC regulates transcription during the S phase, when replication and transcription machineries compete for the same DNA template and can therefore interfere with each other and cause DNA damage. DRC temporarily downregulates transcription by RNAPII and RNAPIII during encounters of transcription and replication machineries. DRC activation during replication stress triggers the disassembly of the preinitiation complexes at tRNA genes^20^ and removes RNA polymerases from chromatin^21,22^. Genotoxic stress does not trigger transcriptional changes only in the vicinity of DNA damage. DDC/DRC activation regulates transcription of specific groups of co-regulated genes by phosphorylating transcription factors, independently of where in the genome the site(s) of DNA damage are located^23–28^.

An interesting and incompletely understood aspect of transcriptional response to genotoxic stress is global inhibition of transcription that affects the expression of genes encoded by DNA that was not damaged by genotoxic stress^9,11,12^. The immediate transcriptional response to UV exposure is global inhibition of transcription elongation^29,30^, followed by inhibition of transcription initiation^30–32^. An important event in response to genotoxic stress and genome-wide transcription shutdown is the regulation of Rpb1p subunit of RNAPII by ubiquitination at K_1268_ (K_1246_ in yeast) and degradation^19^. However, it is not known whether this is the only mechanism of global inhibition of transcription after genotoxic stress.

The likely purpose of the global inhibition of transcription by RNAPI, RNAPII, and RNAPIII is to make DNA accessible for repair, avoid transcription of damaged genes, and conserve cellular resources; however, the mechanisms are largely unknown. Our results show that a relatively brief treatment with HU globally inhibits RNA synthesis and transcription by RNAPI, RNAPII, and RNAPIII. HU inhibits transcription termination and pre-mRNA cleavage at the pA sites, destabilizes mRNAs, and shortens poly(A) tails of mRNAs, indicating a defect in pre-mRNA 3′ end processing. Interestingly, inactivation of the checkpoint kinase Mec1p downregulates the efficiency of transcription termination and reduces the efficiency of pre-mRNA clevage at the pA sites, suggesting the involvement of DNA damage checkpoint in transcription termination and pre-mRNA 3′ end processing.

## Results

### HU globally inhibits RNA synthesis

HU is an inhibitor of ribonucleotide reductase, decreases dNTP levels and slows down the progression of replication forks, resulting in activation of DRC. HU does not directly damage DNA; however, stalled replication forks occasionally collapse or break, causing secondary DNA damage. Since yeast can grow in the presence of 200 mM HU, it is likely that a 30 min HU treatment does not cause extensive DNA damage and the transcriptional responses to HU are due to activation of the checkpoint kinases of DRC^33,34^.

**Figure 1:**
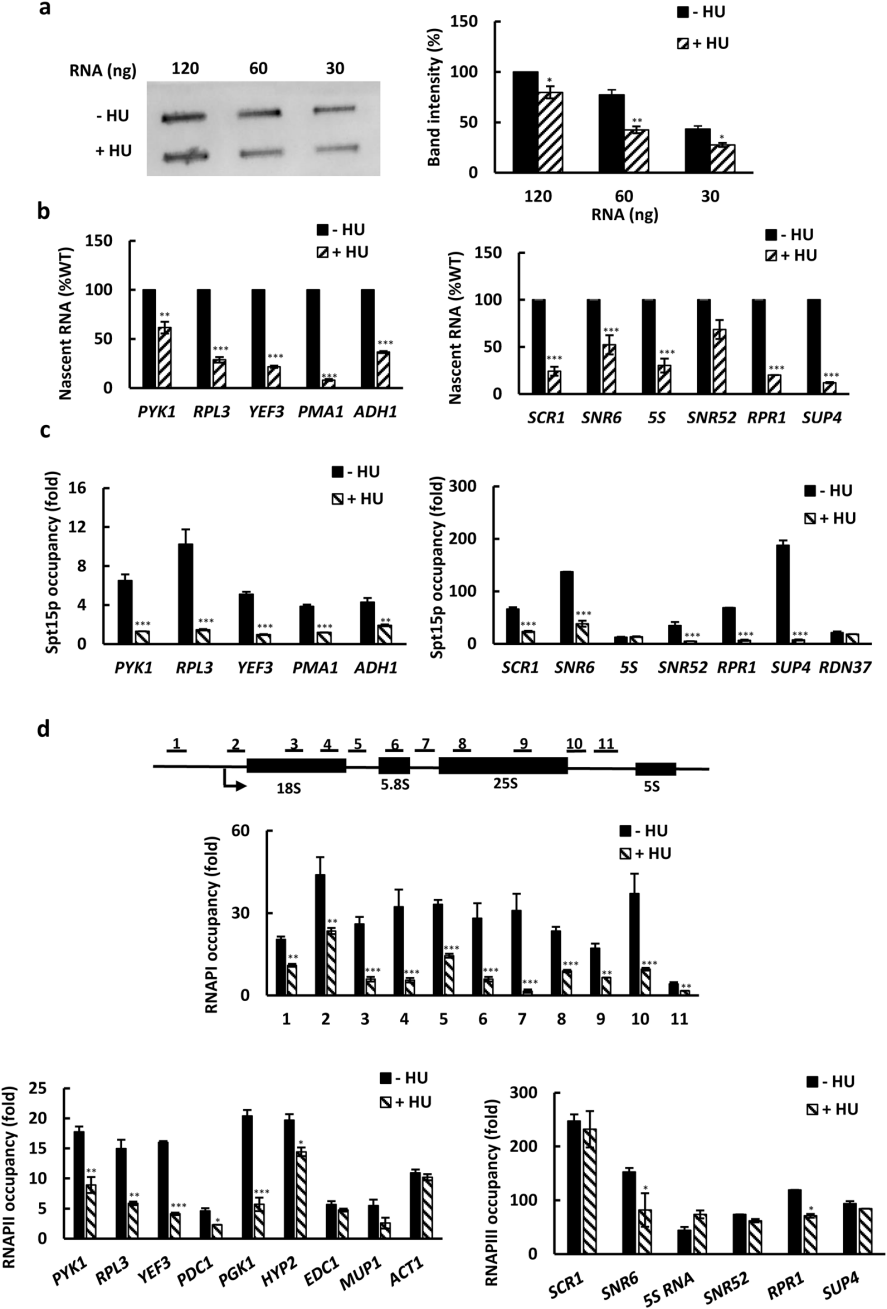
HU globally inhibits RNA synthesis and transcription by RNAPI, II, and III. (**a**) Slot blot analysis of RNA isolated from WT cells (W303-1a) before and after treatment with 200 mM hydroxyurea (HU) for 30 min. The figure represents typical results from three biologically independent experiments. The right panel shows quantitative analysis of the slot blot. The intensity of each band was quantified by densitometry and the results were normalized to untreated WT cells*.* (**b**) Nascent RNA levels of genes transcribed by RNAPII (left panel) and RNAPIII (right panel) in WT cells before and after treatment with 200 mM HU for 30 min. The results are expressed relative to the value for the WT strain and are normalized to *S. pombe* tubulin mRNA. The experiments were repeated three times, and the results are shown as the means ± SD. (**c**) Occupancies of Spt15p in WT cells expressing *SPT15* tagged with three copies of the HA epitope (strain AD066) before and after treatment with 200 mM HU for 15 min at genes transcribed by RNAPII (left panel) and RNAPI and RNAPIII (right panel). (**d**) Occupancies of RNAPI (strain JS311-A190MN), RNAPII (strain W303-1a), and RNAPIII (strain RET1-13MYC) at highly transcribed genes before and after treatment with 200 mM HU for 15 min. (**c**, **d**) Each immunoprecipitation was performed at least three times using different chromatin samples, and the occupancy at the indicated genes was calculated using the *POL1* coding sequence as a negative control. The data are presented as fold occupancy over the *POL1* coding sequence control and represent the means ± SD. (**a**–**d**) Three biologically independent experiments were analyzed by Mann–Whitney test using GraphPad Prism 9.5.1. Values for HU-treated samples that are statistically different from values for untreated samples are indicated: *p < 0.05; **p < 0.01; and ***p < 0.001.

To determine whether treatment with HU globally inhibits RNA synthesis in *Saccharomyces cerevisiae*, cells were grown in rich YPD medium, treated with 200 mM HU for 30 min, and subsequently pulse-labeled with 4-thiouracil (4tU). In vivo pulse labeling of RNA with 4tU for a short period of time and quantification of the labeled RNA provides a convenient readout of the frequency of transcription. Total RNA was isolated, labeled with biotin, and analyzed by slot blot analysis with streptavidin–horseradish peroxidase detection. The results indicate that treatment with HU reduced total RNA synthesis by about 30% (Fig. 1a).

Since HU inhibits transcription of ribosomal RNA by RNAPI^13^, we wanted to determine whether HU also affects RNA synthesis by RNAPII and RNAPIII. For this analysis, cells were pulse-labeled with 4tU and mixed in a fixed ratio with labeled *S. pombe* cells for normalization. RNA was isolated, biotinylated, and nascent RNA was isolated on streptavidin-coated magnetic beads. The RNAPII transcripts of *PYK1*, *RPL3*, *YEF3*, *PMA1*, and *ADH1*, and RNAPIII transcripts of *SCR1*, *SNR6*, 5S RNA, *SNR52, RPR1*, and *SUP4* in the nascent RNA were quantified by reverse transcription-quantitative PCR (RT-qPCR) (Fig. 1b). All examined transcripts were reduced by the HU treatment to levels ranging from about 60% for *PYK1* to less than 10% for *PMA1* in comparison with the untreated samples (Fig. 1b).

To determine whether HU treatment affects the recruitment of the transcription machinery to the promoters of genes transcribed by RNAPI, II, III, we determined the occupancy of Spt15p, the yeast TATA-binding protein, at the corresponding promoters (Fig. 1c). In addition, we determined the occupancy of Rpa190p, the largest subunit of RNAPI, over the ribosomal RNA gene *RDN37*, occupancy of Rpb1p, the largest subunit of RNAPII, in the promoter regions of several genes transcribed by RNAPII, and occupancy of Ret1p, the second largest subunit of RNAPIII, over several genes transcribed by RNAPIII (Fig. 1d). The results reveal that HU does not uniformly inhibit recruitment of Spt15p to RNAPI, RNAPII, and RNAPIII promoters. While the occupancies of Spt15p in the promoters of RNAPI-transcribed *RDN37* and RNAPIII-transcribed *5S* ribosomal RNA genes were not affected by HU, the occupancies of Spt15p at all examined RNAPII promoters and the remaining RNAPIII promoters were reduced by HU (Fig. 1c). The occupancy of Rpa190p was significantly reduced throughout the ribosomal RNA gene coding region. The occupancies of Rpb1p in the promoter-proximal regions of protein coding sequence (CDS) of RNAPII genes were reduced, but to different levels. HU caused a significant reduction of Rpb1p occupancy in all tested genes except *EDC1* and *ACT1*. Except for *SNR6* and *RPR1*, the Ret1p occupancy was not reduced at any of the examined RNAPIII genes (Fig. 1d).

### Transcriptional effect of HU is attenuated in checkpoint mutants and by altered chromatin structure

**Figure 2:**
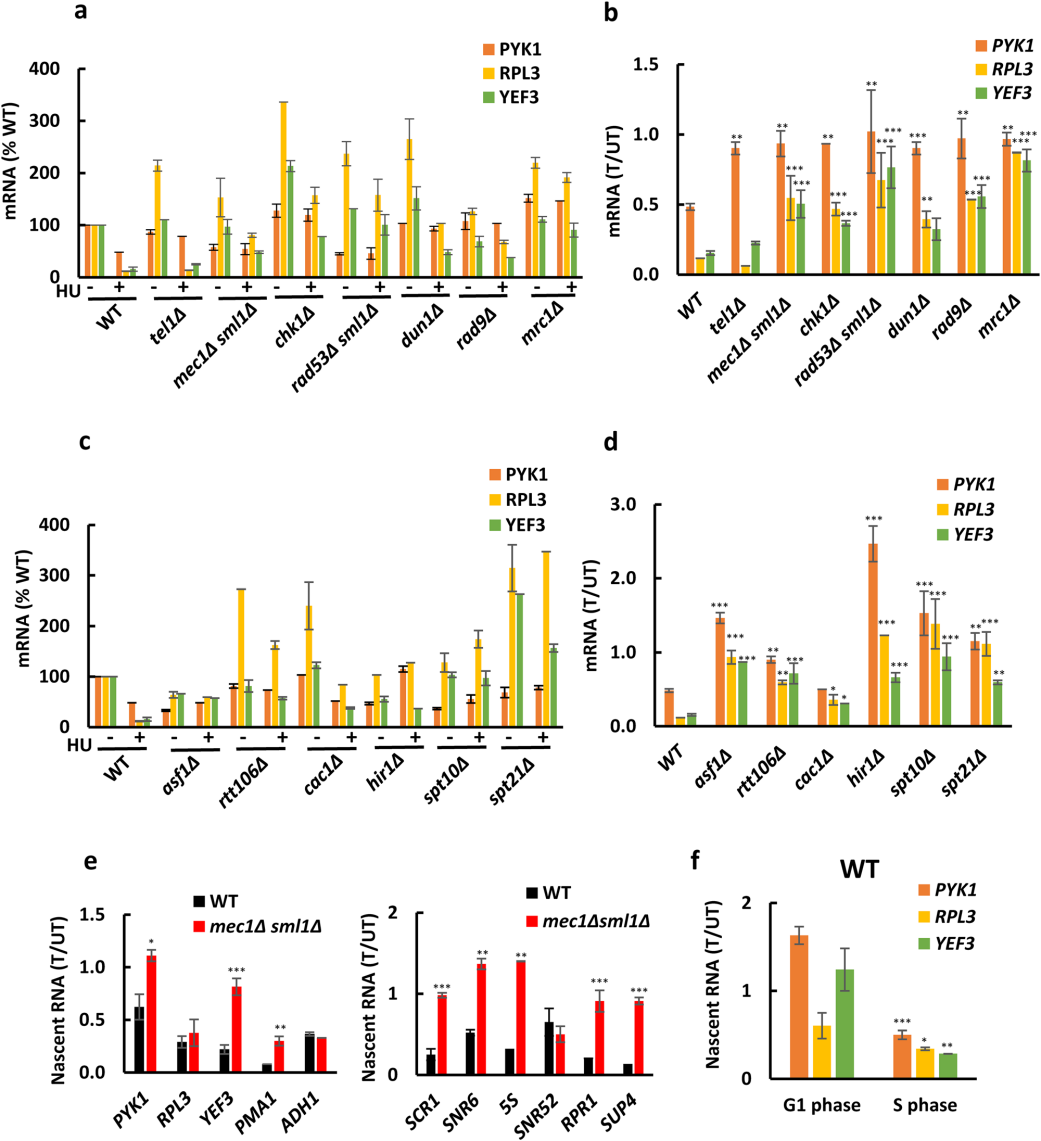
The transcriptional effect of HU is attenuated in checkpoint and chromatin mutants. *PYK1*, *RPL3*, and *YEF3* mRNA levels in WT (W303-1a), *tel1*Δ (SN159), *mec1Δsml1*Δ (SN117), *chk1*Δ (SN136), *rad53Δsml1*Δ (LG606), *dun1*Δ (SN141), *rad9*Δ (SJ027) and *mrc1*Δ (SJ015) cells (**a**) and WT (W303-1a), *rtt106*Δ (MZ642), *rtt109*Δ (MZ655), *hir1*Δ (MZ700), *cac1*Δ (MZ553), and *asf1*Δ (MZ576) cells (**c**) before and after treatment with 200 mM HU for 30 min. The results are expressed relative to the value for the WT strain and were normalized to *RDN25* RNA. (**b, d**) mRNA levels remaining after the HU treatment, calculated as a ratio of mRNA levels in treated (T)/untreated (UT) samples. (**e**) Nascent mRNA levels remaining after treatment with 200 mM HU for 30 min, calculated as a ratio of nascent mRNA levels in treated (T)/untreated (UT) samples for WT (W303-1a) and *mec1*Δ*sml1*Δ (SN117) cells. (**f**) WT (W303-1a) cells were arrested with α-factor in G1-phase, divided in 2 aliquots, and one aliquot treated for 30 min with HU for the last 30 min of the α-factor arrest. The HU treatment of S-phase cells was performed by arresting WT (W303-1a) cells with α-factor and releasing them into YPD medium or YPD medium containing HU for 30 min. The results were normalized to *S. pombe* tubulin mRNA. (**a**–**f**) The experiments were repeated three times, and the results are shown as the means ± SD. (**b**, **d**, **e**) Results from three biologically independent experiments were analyzed by Mann–Whitney test using GraphPad Prism 9.5.1. Values for mutant strains that are statistically different from values for WT strain are indicated: *p < 0.05; **p < 0.01; and ***p < 0.001. (**f**) Values for S phase cells that are statistically different from values for G1 phase cells are indicated: *p < 0.05; **p < 0.01; and ***p < 0.001.

The effect of HU on transcription would be expected to require the checkpoint kinases and occur during the S phase. To test this prediction, we determined *PYK1*, *RPL3*, and *YEF3* mRNA levels before and after HU treatment (200 mM HU for 30 min) in wild-type (WT) cells and cells with inactivated checkpoint kinases *mec1*Δ*sml1*Δ, *tel1*Δ*, rad53*Δ*sml1*Δ, *chk1*Δ, and *dun1*Δ cells; *mec1*Δ and *rad53*Δ cells are viable only if harboring the *sml1*Δ mutation^35^ (Fig. 2a). We used these values to calculate fractions of *PYK1*, *RPL3*, and *YEF3* remaining after the HU treatment (Fig. 2b). In WT cells, the level of repression varied from about 50% for *PYK1*, to about 10% for *RPL3* (Fig. 2b). The HU-mediated repression was most significantly attenuated in *mec1*Δ*sml1*Δ, *rad53*Δ*sml1*Δ, and *mrc1*Δ cells. This result is consistent with the notion that the effect of HU on transcription requires activation of DRC.

The extent of the HU-mediated reduction in RNA levels differed significantly amongst individual genes, with *PYK1* being relatively immune to this reduction and *RPL3* and *YEF3* being significantly more responsive (Fig. 2b). This can be at least partly explained by the differences in the stabilities of the corresponding mRNA. *PYK1* mRNA is significantly more stable than *RPL3* or *YEF3* mRNAs^36^. In general, steady-state levels of mRNAs with greater stability, such as *PYK1*, would be expected to be affected less by 30 min of HU treatment.

The HU-mediated repression of histone genes is attenuated in strains with deletions of histone chaperones^37^. To find out if histone chaperones or other factors required for chromatin assembly and architecture are required for HU-mediated reduction in RNA levels, we determined *PYK1*, *RPL3*, and *YEF3* mRNA levels in WT, *asf1*∆, *rtt106*∆, *cac1*∆, *hir1*∆, *spt10*∆, and *spt21*∆ cells before and after 30 min exposure to HU (Fig. 2c) and calculated fractions of *PYK1*, *RPL3*, and *YEF3* remaining after the HU treatment (Fig. 2d). While *ASF1, RTT106, CAC1,* and *HIR1* encode histone chaperones, *SPT10* and *SPT21* encode transcription factors specific for histone genes. The results showed that the HU effect on RNA levels was decreased by inactivation of all of the tested chromatin factors (Fig. 2d). The easiest interpretation of this result is that the altered chromatin structure in these mutants permits transcription in the presence of HU. However, this interpretation is complicated by the fact that a variety of factors, including histone chaperones, are required for suppression of transcriptional initiation from within coding regions^38–42^. Thus, this cryptic transcription may be at least partly responsible for the suppression of the HU-mediated transcriptional repression in mutants with altered chromatin structure.

The steady-state level of RNA is determined by its synthesis and degradation rates. To assess the effect of HU on the frequency of transcription, we measured nascent RNA in WT and *mec1*Δ*sml1*Δ cells before and after HU treatment (Fig. 2e). The results were in agreement with the measurements of total RNA and showed attenuated HU-mediated reduction in RNA levels in *mec1*Δ*sml1*Δ cells in comparison with WT cells not only for RNAPII transcripts *PYK1, RPL3, YEF3,* and *PMA1*, but also for RNAPIII transcripts *SCR1*, *SNR6*, *5S RNA*, *RPR1*, and *SUP4* (Fig. 2e).

Since the DRC is activated during the S phase, we compared the extent of the HU-mediated reduction in RNA levels in WT cells arrested in G1 phase with WT cells released into S phase (Fig. 2f). Cells were arrested with α-factor, divided in 2 aliquots, and one aliquot treated for 30 min with HU for the last 30 min of the α-factor arrest. In a separate experiment, cells arrested in G1 phase were released into S phase for 30 min in YPD medium or YPD medium containing HU as described^25,26^. The cell cycle arrest in G1 phase and release into S phase were monitored by flow cytometry. The results showed that cells in G1 phase are significantly less responsive to transcription repression by HU than S phase cells (Fig. 2f).

### HU removes RNAPII from chromatin in a gene-specific manner

**Figure 3:**
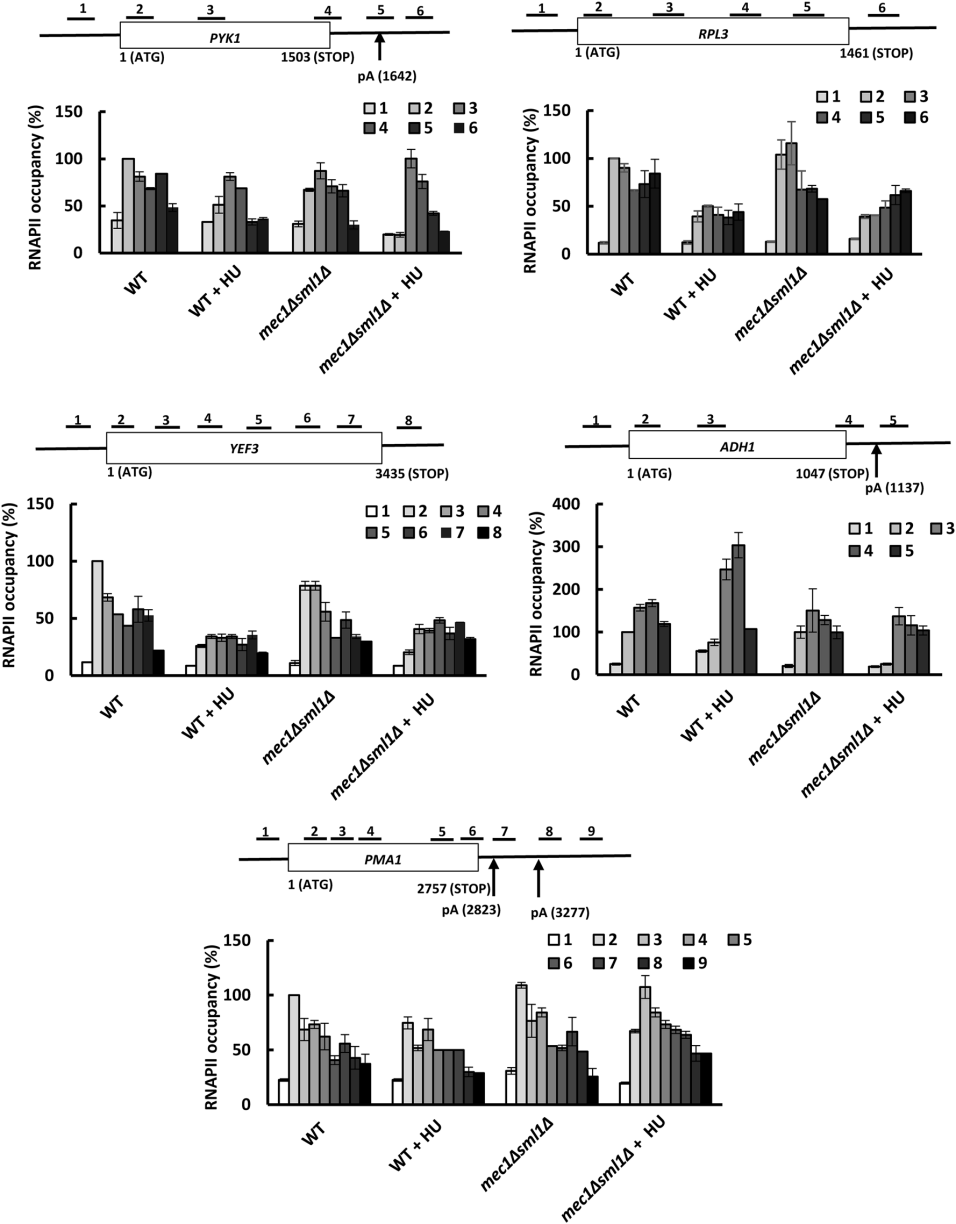
HU removes RNAPII from chromatin in a gene-specific manner. Occupancies of RNPII across *PYK1*, *RPL3*, *YEF3*, *PMA1*, and *ADH1* genes before and after treatment with 200 mM hydroxyurea (HU) for 15 min in WT (W303-1a, SN691) and *mec1*Δ*sml1*Δ (SN117) cells. The top diagram of each gene shows schematic representation of the primers used in ChIP analysis. The positions of the polyadenylation sites (pA) in *PYK1*, *PMA1*, and *ADH1* are according to^43,44^. Each immunoprecipitation was performed at least three times using different chromatin samples, and the occupancy at the indicated genes was calculated using the *POL1* coding sequence as a negative control. The data are presented as a percentage relative to the occupancy at the beginning of the CDS (primer 2 for all genes) for the WT strain and represent the means ± SD.

Our results showed that HU reduces occupancy of Rpb1p, the largest subunit of RNAPII, in the promoter-proximal regions of CDS (Fig. 1d). To determine whether HU-mediated RNAPII removal from chromatin occurs evenly throughout CDS, we determined Rpb1p occupancies within the promoters and coding regions of *PYK1, RPL3, YEF3, PMA1,* and *ADH1* genes in WT and *mec1*∆*sml1*∆ cells before and after treatment with HU (Fig. 3). Comparison of the profiles allows several conclusions. First, HU does not considerably affect processivity of RNAPII. Second, the RNAPII occupancies in WT and *mec1*∆*sml1*∆ cells before HU treatment did not substantially differ in any of the tested genes. Third, and rather surprisingly, HU appreciably decreased the RNAPII occupancies throughout the CDS of only *RPL3* and *YEF3* genes, while the RNAPII occupancies within the CDS of *PYK1*, *PMA1*, and *ADH1* genes in WT cells were not considerably reduced (Fig. 3). This finding contrasts with significantly reduced nascent mRNA levels of all three genes after HU treatment (Fig. 1b). Fourth, HU decreased RNAPII occupancies at 5′ ends of CDSs for all genes in both WT and *mec1*∆*sml1*∆ cells. Fifth, in many positions throughout the CDS of *PMA1, YEF3,* and *RPL3* genes, the RNAPII occupancy after the HU treatment was higher in *mec1*∆*sml1*∆ cells than in WT cells, particularly in the 3′ portions of the CDS and 3′-UTR.

### HU does not trigger cryptic transcription

**Figure 4:**
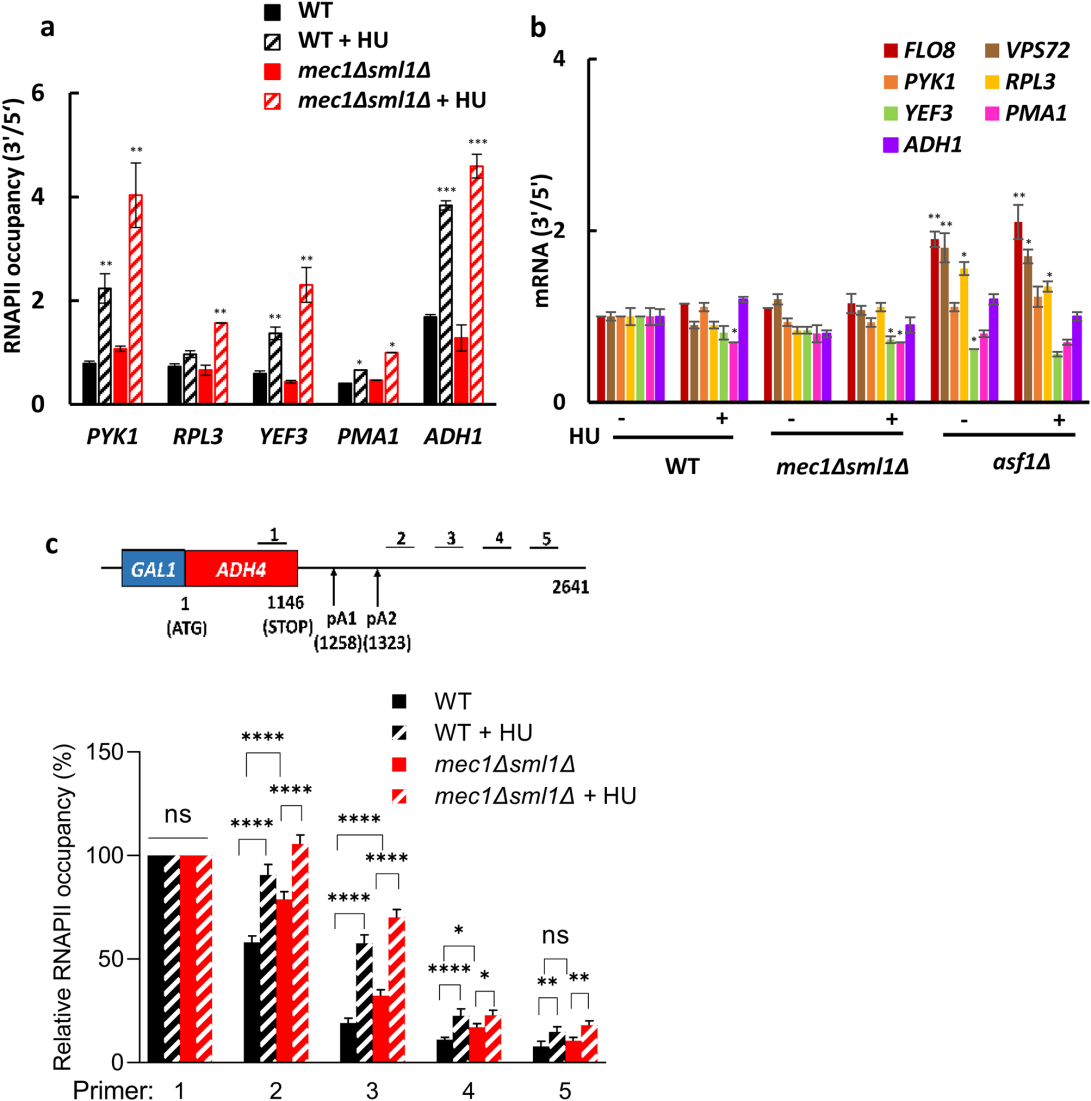
HU and *MEC1* inactivation do not trigger cryptic transcription but reduce efficiency of transcription termination. (**a**) Ratio of RNAPII occupancies at 3′ and 5′ ends of *PYK1*, *RPL3*, *YEF3*, *PMA1*, and *ADH1* genes before and after treatment with 200 mM hydroxyurea (HU) for 15 min in WT (W303-1a) and *mec1*Δ*sml1*Δ (SN117) cells. The ratios were calculated using values from Fig. 3 that correspond to the following ChIP primer positions in *PYK1* (2, 4), *RPL3* (2, 5), *YEF3* (2, 7), *PMA1* (2, 6), and *ADH1* (2, 4). Three biologically independent experiments were analyzed by Mann–Whitney test using GraphPad Prism 9.5.1. Values for the HU-treated samples that are statistically different from values for the corresponding untreated samples are indicated: *p < 0.05; **p < 0.01; and ***p < 0.001. (**b**) Ratios of mRNA levels at 3′ and 5′ ends of the indicated genes before and after HU treatment in WT (W303-1a), *mec1*Δ*sml1*Δ (SN117) and *asf1*Δ (MZ576) cells. The results were corrected for the efficiency of primers and normalized to *RDN25* RNA. Three biologically independent experiments were analyzed by Mann–Whitney test using GraphPad Prism 9.5.1. Values that are statistically different from values for WT untreated samples are indicated: *p < 0.05; **p < 0.01. (**c**) Transcription termination assay was performed with cells containing *GAL1::ADH4* construct. The top diagram shows schematic representation of the *GAL1::ADH4* construct and positions of primers used in ChIP analysis. The positions of the polyadenylation (pA) sites are indicated. RNAPII occupancies across CDS and 3′-UTR of *ADH4* with and without HU (200 mM, 30 min) in WT (DBY 548) and *mec1*Δ*sml1*Δ (RM168) cells were analyzed by ChIP. The data from three biologically independent experiments are presented as fold occupancy over the *POL1* coding sequence control and were calculated as a percentage relative to the occupancy at position 1 (CDS) and represent the means ± SD. The data for each primer were analyzed by two-way ANOVA with Tukey’s correction for multiple comparisons using GraphPad Prism 9.5.1; *p < 0.05; **p < 0.01; ***p < 0.001.

The observation that Rpb1p occupancy after the HU treatment was higher at the 3′ portions of the CDS and 3′UTR of *PMA1, YEF3,* and *RPL3* in *mec1*∆*sml1*∆ than in WT cells can be explained by at least two mutually non-exclusive mechanisms. The first possibility is that HU triggers cryptic transcription, and this effect is exacerbated in *mec1*∆*sml1*∆ cells. The second possibility is that HU treatment causes a defect in transcriptional termination and perhaps RNAPII stalling within the 3′ portions of CDS and 3′-UTR, particularly in *mec1*∆*sml1*∆ cells. One of the hallmarks of cryptic transcription is elevated Rpb1p occupancy at the 3′ ends of CDS in comparison with the 5′ ends. To test whether treatment with HU leads to inappropriate transcriptional initiation within coding regions, we evaluated the ratios of Rpb1p occupancies at the 3′ and 5′ ends of the CDS in *PYK1, RPL3, YEF3, PMA1,* and *ADH1* genes in WT and *mec1*∆*sml1*∆ cells before and after treatment with HU. The 3′/5′ ratios of Rpb1p occupancies do not significantly differ between WT and *mec1*∆*sml1*∆ cells (Fig. 4a). Except for *RPL3*, HU treatment of WT cells elevated the Rpb1p 3′/5′ ratios for other tested genes, and this increase was significantly more pronounced in *mec1*∆*sml1*∆ cells (Fig. 4a).

To test whether the elevated Rpb1p 3′/5′ ratios observed in WT and *mec1*∆*sml1*∆ cells after HU treatment really reflect inappropriate transcriptional initiation within coding regions, we determined mRNA levels in wild-type and *mec1*∆*sml1*∆ strains at the 5′ and 3′ portions of CDS of *PYK1, RPL3, YEF3, PMA1,* and *ADH1* genes (Fig. 4b). As a positive control, we included *asf1*∆ strain, known to exhibit cryptic transcription and elevated 3′/5′ mRNA ratios of number of genes, including *FLO8* and *VPS72*^38–42,45^. To make the mRNA levels at the 5′ and 3′ end of each gene comparable, we corrected the results for the efficiency of primers. Thus, in the absence of cryptic transcription or premature RNAPII termination, the 3′/5′ ratios are expected to be very close to 1. As expected, the 3′/5′ mRNA ratios of *FLO8* and *VPS72* genes were elevated in *asf1*∆ strain and these ratios were not exacerbated by the HU treatment (Fig. 4b). Treatment of WT or *mec1*∆*sml1*∆ cells with HU did not elevate the 3′/5′ mRNA ratios for any of the examined genes. Since suppression of cryptic transcription within genes requires normal histone levels and function of a number of chromatin regulators^42^, this result suggests that *MEC1* inactivation or HU treatment of wild type or *mec1*∆*sml1*∆ cells do not result in gross chromatin changes that would lead to widespread cryptic transcription. In addition, HU treatment of wild-type and *mec1*∆*sml1*∆ cells slightly decreased the 3′/5′ mRNA ratios for only *PMA1*, *YEF3* and *PMA1*, respectively. This result suggests that HU does not trigger a widespread premature RNAPII termination^46^ (Fig. 4b).

### HU treatment or *MEC1* inactivation decrease efficiency of transcription termination

The RNAPII occupancies within CDSs and 3′-UTRs of *PYK1, RPL3, YEF3, PMA1,* and *ADH1* genes (Fig. 3) do not allow conclusion whether HU or *MEC1* inactivation affect transcription termination. The likely reason is that the *Saccharomyces cerevisiae* genome is tightly packed and the intergenic regions are small, making it difficult to resolve transcription termination of one gene from transcription events at adjacent genes by conventional ChIP. To circumvent this problem, we used strains expressing *GAL1-ADH4* construct (47). Transcription of the chromosomal *ADH4* gene in this reporter construct is driven by a strong inducible *GAL1* promoter. The advantage of this construct is that there are no transcription units within 4.7 kb of 3′-UTR of *ADH4* and the transcription termination of *ADH4* can be analyzed in the absence of any interference from neighboring genes (47). Our experiments showed that both HU and *MEC1* inactivation reduce the efficiency of transcription termination as indicated by the elevated RNAPII occupancies downstream of the pA sites in the 3′-UTR of *ADH4* (Fig. 4c).

### HU treatment or *MEC1* inactivation decrease processing efficiency at polyadenylation (pA) sites

**Figure 5:**
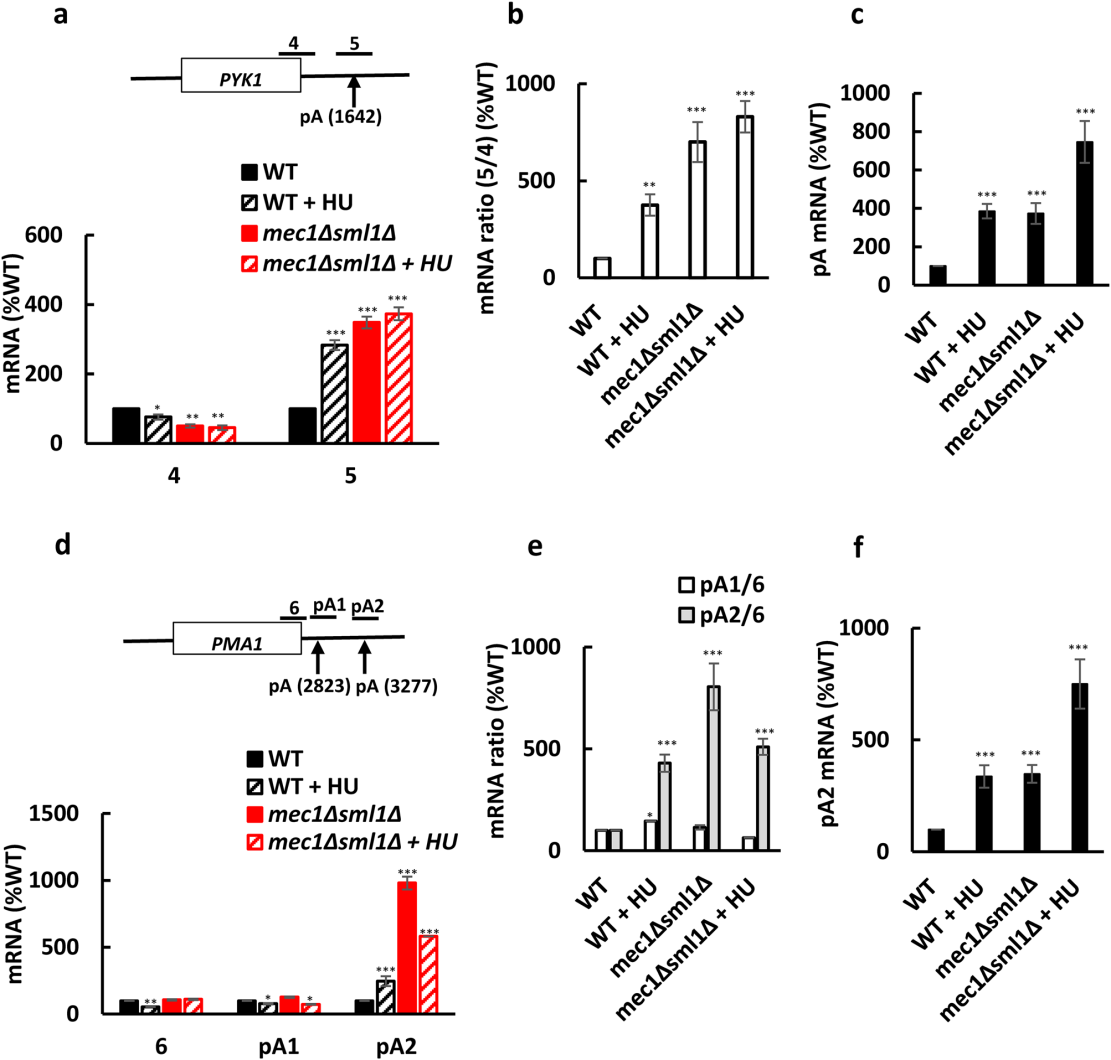
HU and *MEC1* inactivation reduce processing efficiency at pA sites. (**a**–**c**) HU and *MEC1* inactivation reduce processing efficiency at the *PYK1* pA site. (**a**) mRNA levels at the indicated positions of *PYK1*. The results for each primer are normalized to *RDN25* RNA and expressed relative to the value for the WT strain. (**b**) Ratios of mRNA levels at positions 5 and 4. The results are expressed relative to the value for the WT strain. (**c**) Strand-specific reverse transcription quantitative PCR at the *PYK1* pA site. Total RNA was reversely transcribed using PYK1-5 reverse primer and the cDNA spanning the pA site was quantified by qPCR with PYK1-5 forward and reverse primers. The results are expressed relative to the value for the WT strain and are normalized to *RDN25* RNA. (**d**–**f**) HU and *MEC1* inactivation reduce processing efficiency at the *PMA1* pA2 site. (**d**) mRNA levels at the indicated positions of *PMA1*. The results for each primer are normalized to *RDN25* RNA and expressed relative to the value for the WT strain. (**e**) pA1/6 and pA2/6 mRNA ratios. The results are expressed relative to the value for the WT strain. (**f**) Strand-specific reverse transcription quantitative PCR at the *PMA1* pA2 site. Total RNA was reversely transcribed using PMA1-pA2 reverse primer and the cDNA spanning pA2 site was quantified by qPCR with PMA1-pA2 forward and reverse primers. Three biologically independent experiments were analyzed by Mann-Whitney test using GraphPad Prism 9.5.1. Values that are statistically different from values for untreated WT strain are indicated: *p < 0.05; **p < 0.01; and ***p < 0.001.

After RNAPII transcribes over the polyadenylation (pA) site, the pre-mRNA is cleaved at the pA site by the multi-component complexes cleavage and polyadenylation factor (CPF) and cleavage factor (CF), consisting of cleavage factors IA and IB (CFIA and CFIB)^48–50^. The CPF subunit Ysh1p/Brr5p is the endonuclease that cleaves the nascent RNA. To address whether inactivation of *MEC1* and/or HU treatment of WT or *mec1*∆*sml1*∆ cells affect the efficiency of cleavage at the pA site of *PYK1* pre-mRNA, we measured the level of pre-mRNA transcripts that were not cleaved at the pA site by RT-qPCR with primer pair that spans the pA site (Fig. 5a). To account for the differences in the pre-mRNA levels between WT and *mec1*∆*sml1*∆ cells and due to the HU treatment, we normalized the results with pre-mRNA levels upstream of the pA site (Fig. 5b). The results showed that the efficiency of *PYK1* pre-mRNA cleavage at the pA site is reduced in *mec1*∆*sml1*∆ cells, and also after the HU treatment (Fig. 5a,b). To eliminate the possibility that these results are affected by the presence of anti-sense non-coding transcript, we quantified the unprocessed pre-mRNA by strand-specific reverse transcription (RT) and qPCR. The total RNA was reverse transcribed with the primer complementary to the *PYK1* pre-mRNA downstream of the pA site and the cDNA generated from the pre-mRNA not cleaved at the pA site was quantified by qPCR with primer pair that spans the pA site. These results consistently indicate that HU or *MEC1* inactivation reduce the efficiency of *PYK1* pre-mRNA cleavage at the pA site (Fig. 5c). We performed a similar analysis for *PMA1* gene that contains two pA sites. Our results show that HU or *MEC1* inactivation reduce the efficiency of *PMA1* pre-mRNA cleavage at the pA2, but not at the pA1 site (Fig. 5d,e). Reduced processing efficiency in *mec1*∆*sml1*∆ cells and due to the HU treatment at the *PMA1* pA2 site was confirmed by strand-specific RT and qPCR (Fig. 5f). We conclude that both HU treatment or *MEC1* inactivation reduce processing efficiency at the pA sites.

### The transcriptional effect of HU is attenuated in *RAT1* and *XRN1* mutants

**Figure 6:**
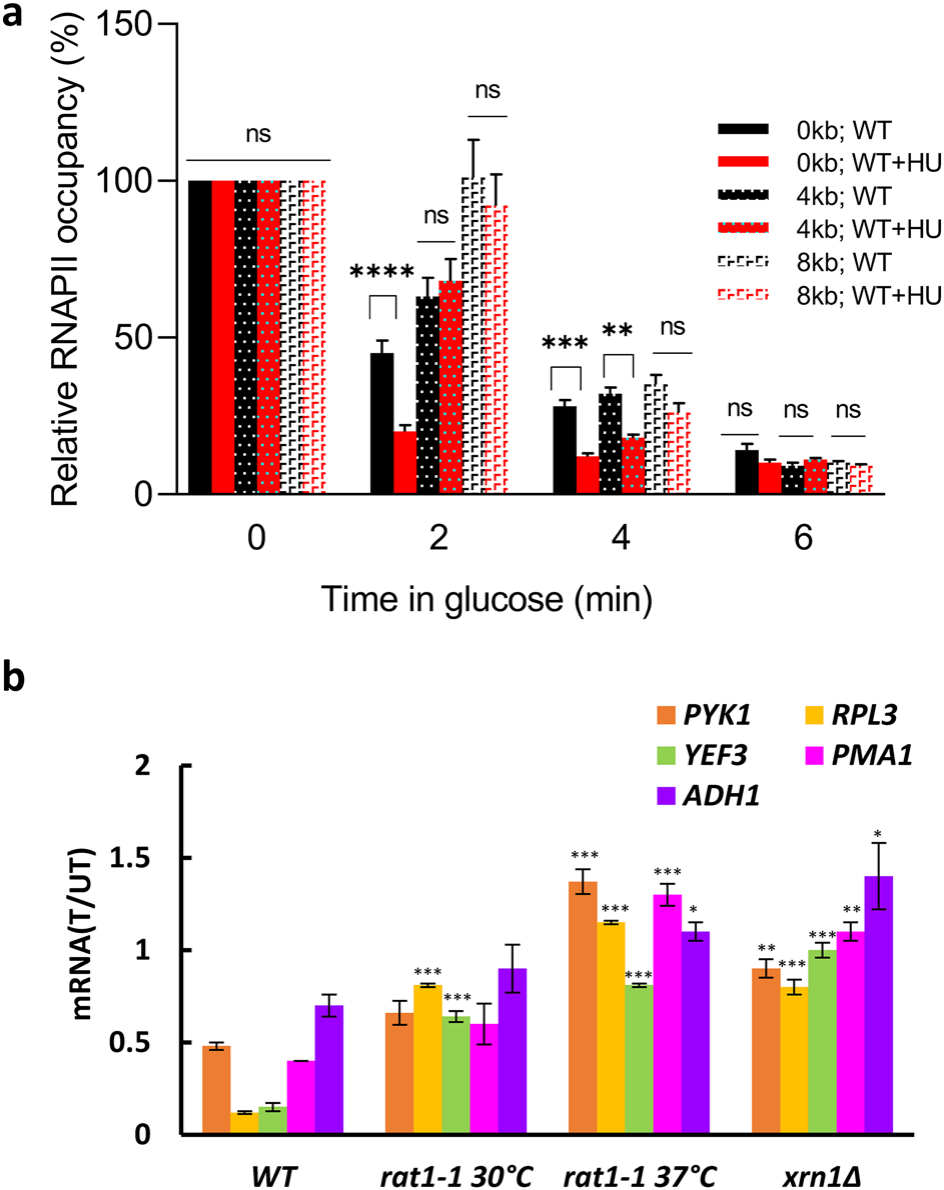
The transcriptional effect of HU is attenuated in *RAT1* and *XRN1* mutants. (**a**) Transcription elongation rate assay. WT cells containing *GAL1::YLR454* construct (SN691) were grown in YP medium with 2% raffinose to an A_600nm_ ~ 0.4 and then induced with 2% galactose for 2 h. Subsequently, 2% glucose was added to stop transcription and RNAPII occupancies were determined after 0, 2, 4, and 6 min at 0, 4, and 8 kb. Each immunoprecipitation was performed at least three times using different chromatin samples, and the occupancy at the indicated positions was calculated using the *POL1* coding sequence as a negative control. The data was calculated as a percentage relative to the occupancy at 0 min and represent the means ± SD. The data for each primer were analyzed by two-way ANOVA with Tukey’s correction for multiple comparisons using GraphPad Prism 9.5.1; *, *p* < 0.05; **, *p* < 0.01; ***, *p* < 0.001. (**b**) mRNA levels remaining after 30 min HU treatment, calculated as a ratio of mRNA levels in treated (T)/untreated (UT) samples for WT (W303-1a), *rat1-1* (YSB1796) cells grown at 30 °C, *rat1-1* cells grown at 30 °C and shifted to 37 °C for 1 h, and *xrn1∆* (MB115) cells. The results were normalized to *RDN25* RNA and shown as the means ± SD. Three biologically independent experiments were analyzed by Mann-Whitney test using GraphPad Prism 9.5.1. Values that are statistically different from values for WT strain are indicated: *p < 0.05; **p < 0.01; and ***p < 0.001.

We were surprised by the finding that the Rpb1p occupancies in the promoter and entire CDS of *PMA1, PYK1,* and *ADH1* in WT cells are not significantly reduced by HU (Fig. 3), despite a significant decrease in the nascent mRNA levels (Fig. 1b). These results suggest that HU does not affect the number of RNAPII molecules transcribing *PMA1*, *PYK1* and *ADH1* genes, but affects the production of the corresponding mRNAs. The simplest possible explanation would be that HU reduces the rate of transcriptional elongation or perhaps induces irreversible stalling of RNAPII on the DNA template. To test this scenario, we employed the very long *YLR454* gene (8 kb) under the control of galactose-inducible *GAL1* promoter^51^. This reporter construct allows measurements of transcription elongation rate by monitoring Rpb1p occupancies at regularly spaced positions on *YLR454* gene at different time points during the “last wave of transcription” after the promoter shut down by glucose. These experiments showed that HU does not reduce the rate of transcriptional elongation (Fig. 6a). To the contrary, it appears that HU causes faster elongation, since the RNAPII occupancy at 0 kb and 4 kb at 4 min after glucose addition was significantly reduced in HU-treated cells in comparison with untreated cells. Consistently, the RNAPII occupancy at 0 kb at 2 min after glucose addition was also reduced in HU-treated cells. These results are quite surprising and elucidating the underlying mechanism will require further work. However, the results show that the difference in the effect of HU on mRNA synthesis and RNAPII occupancy on *PMA1, PYK1,* and *ADH1* genes cannot be explained by slower RNAPII elongation. The results also show that HU does not cause irreversible stalling of RNAPII, since the RNAPII within the coding region of *YLR454* is able to finish the transcription cycle and completely clear the gene after the promoter shut down by glucose.

Another mechanism to reconcile the difference in the effect of HU on mRNA synthesis and RNAPII occupancy on *PMA1, PYK1,* and *ADH1* genes would be the degradation of the produced mRNAs. To assess the involvement of the two major 5′ to 3′ exonucleases Rat1p and Xrn1p in HU-mediated repression of mRNA synthesis, we calculated fractions of *PYK1*, *RPL3*, *YEF3*, *PMA1*, and *ADH1* mRNAs remaining after 30 min of HU exposure in *xrn1*∆ cells and *rat1-1*^*ts*^ cells grown at 30 °C and exposed for 1 h to 37 °C, restrictive temperature for *rat1-1*^*ts*^ cells (Fig. 6b). The HU-mediated repression was significantly attenuated in *rat1-1* cells at 30 °C as well as 37 °C, and in *xrn1*∆ cells. This result suggests that at least part of the repressive effect of HU on mRNA levels is mediated by mRNA degradation.

### HU destabilizes mRNAs

**Figure 7:**
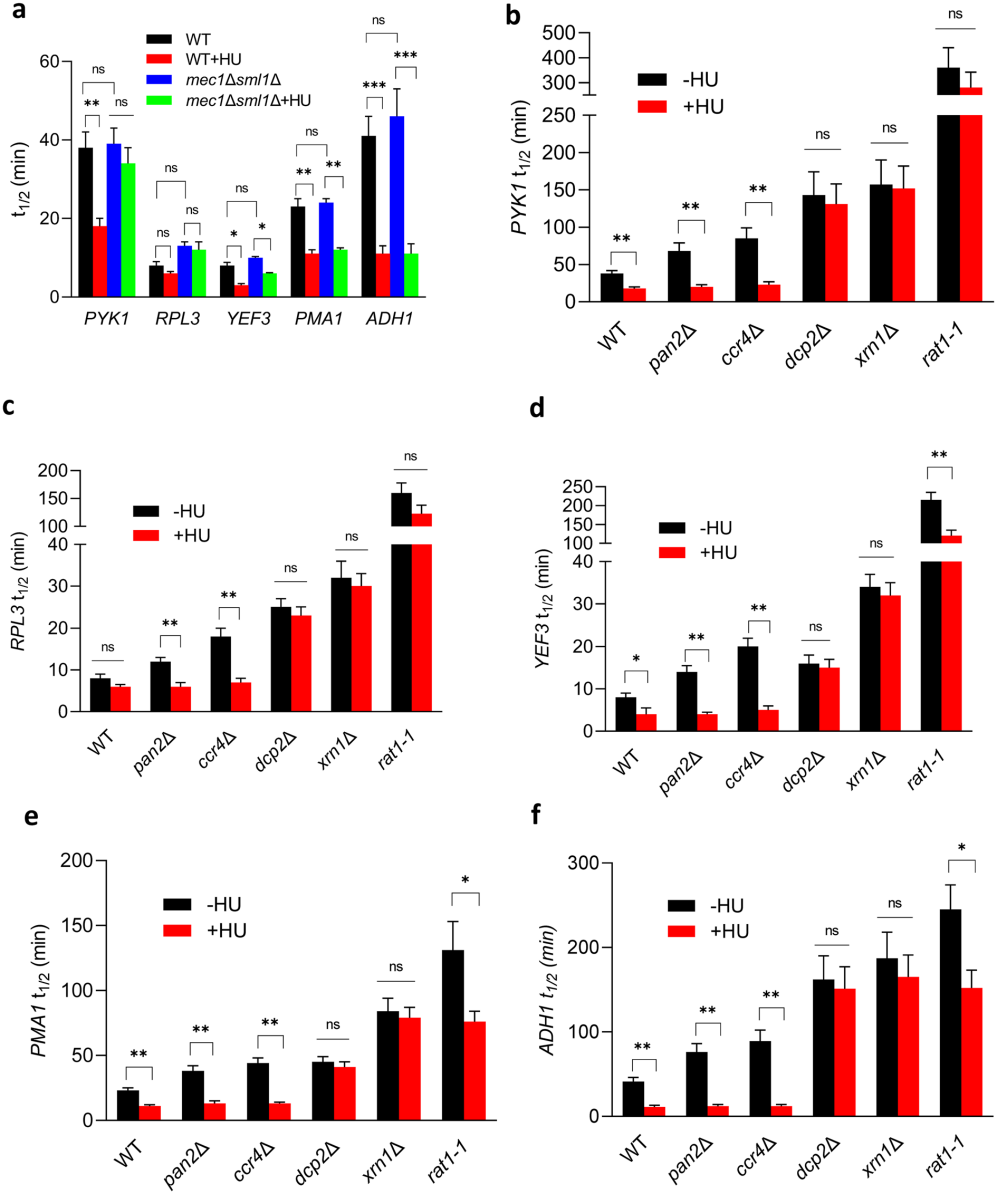
HU destabilizes mRNAs. (**a**) Half-lives of the indicated mRNAs (t_1/2_) in WT (W303-1a) and *mec1*Δ*sml1*Δ (SN117) cells before and after treatment with 200 mM HU for 30 min. Three biologically independent experiments were analyzed by two-way ANOVA with Tukey’s correction for multiple comparisons using GraphPad Prism 9.5.1; *p < 0.05; **p < 0.01; ***p < 0.001. (**b**–**f**) Half-lives of *PYK1*, *RPL3*, *YEF3*, *PMA1*, and *ADH1* mRNA (t_1/2_) in WT (W303-1a), *pan2∆* (MB123), *ccr4∆* (SM096), *dcp2∆* (MB129)*, xrn1∆* (MB115)*,* and *rat1-1∆* (YSB1796) cells before and after the HU treatment. Three biologically independent experiments were analyzed by Mann–Whitney test using GraphPad Prism 9.5.1. Values for HU-treated samples that are statistically different from values for untreated samples in the same strain are indicated: *p < 0.05; **p < 0.01; and ***p < 0.001.

To test directly whether HU treatment affects mRNA stability, we determined half-lives of *PYK1, RPL3, YEF3, PMA1*, and *ADH1* mRNAs in WT and *mec1*∆*sml1*∆ cells before and after HU treatment (Fig. 7a). Transcription was inhibited by thiolutin and mRNA levels were followed by RT-qPCR. *PYK1, RPL3, YEF3, PMA1*, and *ADH1* mRNAs have half-lives within the range of 5 and 50 min^36^, which allows relatively accurate measurements without the need for prolonged incubation with thiolutin. HU treatment destabilized all tested mRNAs in WT cells. The effect of HU on mRNA stability in *mec1*∆*sml1*∆ cells was attenuated for *PYK1* mRNA, while the effect on *YEF3, PMA1*, and *ADH1* mRNAs did not significantly differ from WT (Fig. 7a). We interpret these results to mean that treatment with HU destabilizes some, perhaps many mRNAs in WT cells, and this destabilizing effect of HU for some mRNAs is attenuated in *mec1*∆*sml1*∆ cells. Interestingly, HU does not destabilize histone mRNAs, which are produced only during the S phase and are very unstable with half-lives within 2 and 4 min^37^. Perhaps unstable mRNAs cannot be further destabilized by HU, or destabilization of very short-lived mRNAs cannot be detected by conventional techniques.

The HU-triggered destabilization of mRNAs can be explained by one of the two basic mechanisms. First, HU may activate one or more steps in the mRNA decay pathway, including deadenylation, decapping, and exonucleolytic degradation in 5′ to 3′ and/or 3′ to 5′ direction. Second, HU may alter mRNA processing in a way that makes mRNAs more susceptible to degradation. For example, the nascent mRNAs may not contain the normal cap structure (5′-m^7^GpppN) at the 5′ end or the poly(A) tail at the 3′ end may not be sufficiently long to prevent subsequent exonucleolytic degradation from the 3′ end or decapping and exonucleolytic degradation from the 5′ end. To address these possibilities, we determined the HU effect on *PYK1, RPL3, YEF3, PMA1*, and *ADH1* mRNAs in *pan2*∆, *ccr4*∆, *dcp2*∆, *xrn1*∆, and *rat1-1* cells. Pan2p and Ccr4p are subunits of the Pan2p-Pan3p and Ccr4p-Pop2p-Not deadenylases, respectively. Dcp2p is a subunit of Dcp1p-Dcp2p decapping complex and Xrn1p and Rat1p are two major 5′ to 3′ exonucleases^52^. While HU destabilized all mRNAs in *pan2*∆ and *ccr4*∆ cells to the same extent as in WT cells, HU did not affect mRNA half-lives in *dcp2*∆ and *xrn1*∆ cells and the effect was significantly attenuated in *rat1-1* cells (Fig. 7b-f).

Degradation of mRNAs is initiated by shortening of the poly(A) tail at the 3′ end by the Pan2p-Pan3p and Ccr4p-Pop2p-Not deadenylases (52) and is followed by the removal of the cap structure by the Dcp1p-Dcp2p at the 5′ end. The decapping occurs only after the length of the polyA tail is reduced to 10–12 residues (52–54). Since the HU effect on mRNA stability does not require the Pan2p-Pan3p and Ccr4p-Pop2p-Not deadenylases, but does require the decapping complex Dcp1p-Dcp2p, we conclude that mRNAs produced in the presence of HU contain a normal cap structure at the 5′ end that protects them from decay from the 5′ end, but contain shorter poly(A) tails. Our results are thus consistent with a model in which mRNAs produced in the presence of HU contain shorter poly(A) tails and do not require deadenylation for subsequent decapping and degradation.

### HU shortens poly(A) tails

**Figure 8:**
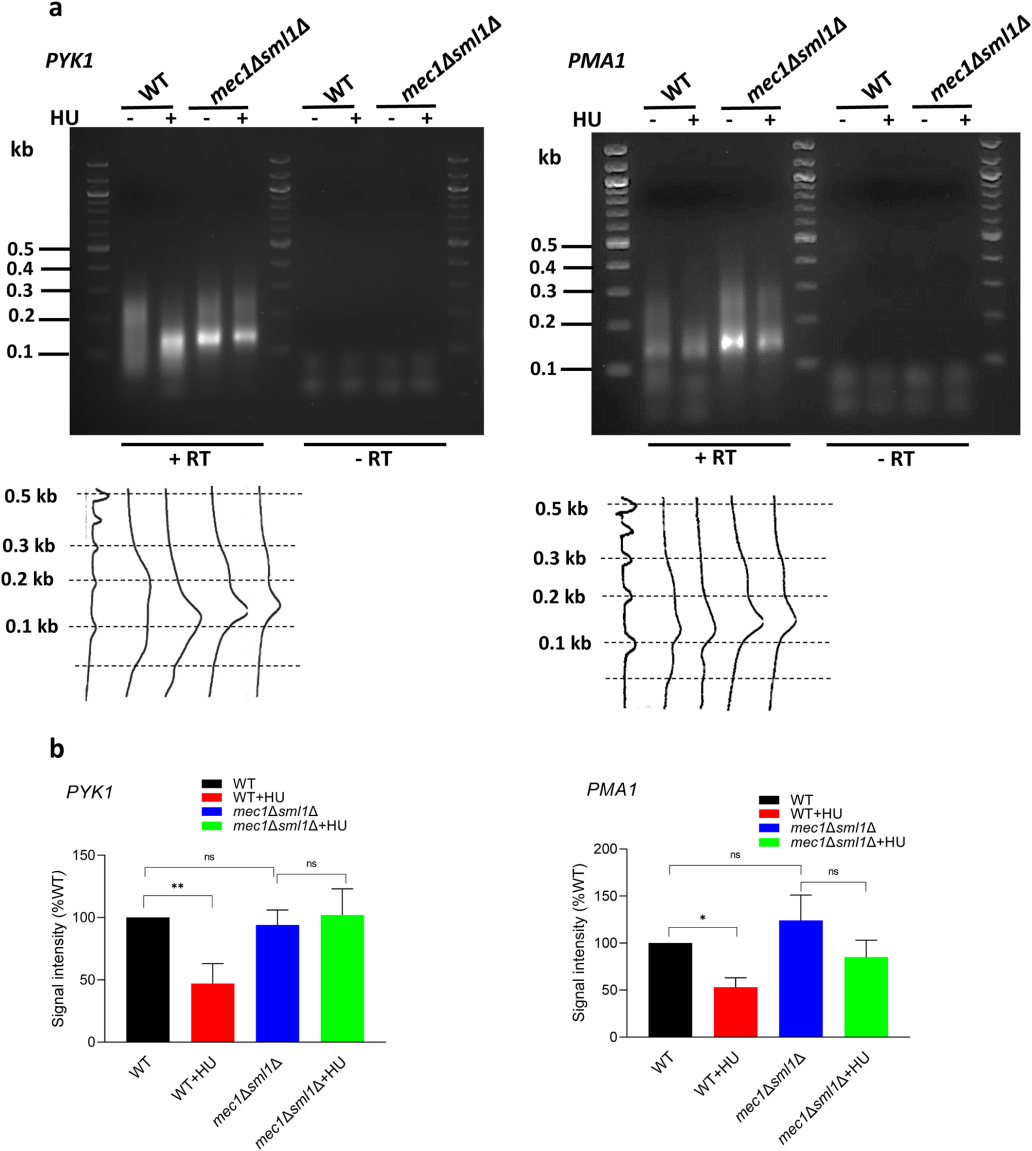
HU shortens poly(A) tails. (**a**) RACE-PAT analysis of poly(A) tail length of *PYK1* (left panel) and *PMA1* (right panel). Total RNA samples from WT and *mec1*Δ*sml1*Δ cells before and after HU treatment were reverse transcribed using pA primer containing oligo(dT) sequence. The resulting cDNAs were amplified by PCR using PYK1-5 forward and pA (*PYK1*) or PMA1-pA2 forward and pA (*PMA1*) primers. The right part of each panel shows a control experiment that omitted reverse transcriptase (-RT). Signal intensities were quantified by densitometry tracing using NIH ImageJ, as shown below each lane in both panels. (**b**) Quantification of signal above 0.2 kb. The data are presented as a percentage of signal above 0.2 kb relative to the total signal in each lane and the results are expressed relative to the value for the WT strain. Three biologically independent experiments were analyzed by one-way ANOVA with Tukey’s correction for multiple comparisons using GraphPad Prism 9.5.1; *p < 0.05; **p < 0.01.

To address directly whether mRNAs produced in the presence of HU contain shorter poly(A) tails, we performed the RACE-PAT (rapid amplification of cDNA ends poly(A) test)^55–58^ on total RNA prepared from WT and *mec1*∆*sml1*∆ cells before and after HU treatment (Fig. 8a). The RNA was reverse-transcribed using a primer containing oligo(dT) sequence and the resulting cDNA was amplified by PCR with this oligo(dT) primer and a gene-specific primer annealing just upstream of the pA site. Since the oligo(dT) primer can hybridize along the entire length of the poly(A) tail, the PCR yields a heterogenous mixture of DNA fragments representing the length of the poly(A) tail. The results appear as a smear on agarose gel: mRNA with longer poly(A) tail yields a smear that corresponds to longer DNA fragments. Quantification of the densitometry tracing indicated that HU treatment of WT cells shortened the poly(A) tail of both *PYK1* and *PMA1* mRNAs, but did not have any statistically significant effect on the length of poly(A) tails of *PYK1* and *PMA1* in *mec1*∆*sml1*∆ cells (Fig. 8a,b). These results suggest that *MEC1* is required for HU-mediated shortening of poly(A) tails in mRNAs.

## Discussion

Perhaps the most surprising and significant finding of this study is that treatment with HU or *MEC1* inactivation causes defects in transcription termination and pre-mRNA 3′ end processing. After the pre-mRNA is cleaved at the pA site by the CPF-CF complexes, the resulting 3′ end of the RNA is polyadenylated by poly(A) polymerase Pap1p, which is recruited to the pre-mRNA by the CPF-CF complexes. The resulting poly(A) tail binds poly(A) -binding proteins, which protect the RNA from exonucleolytic degradation and facilitate nuclear export and translation^59^. The new 5′ end of the RNA created by the cleavage of the nascent transcript is not protected by the cap structure and is degraded by Rat1p exonuclease, which catches up with RNAPII and displaces it from chromatin. Strong evidence indicates that this mechanism, referred to as the torpedo model, cooperates with the allosteric model. The allosteric model posits that the conformation of RNAPII changes after passage of the pA site, most likely due to recruitment of the CPF and CF complexes, and/or loss of elongation factors^47–50,60,61^. In addition to the requirement for the pre-mRNA cleavage at the pA site and transcription termination, the CPF and CF complexes are also required for pre-mRNA polyadenylation^60–63^.

DDR to genotoxic chemicals, including HU, includes degradation of several key subunits of the CPF complex and global inhibition of transcription termination and pre-mRNA 3′-end cleavage and processing in both yeast and human cells^66–69^. However, the relationship between DDR and pre-mRNA 3′ end processing is reciprocal. Mutations in factors involved in transcription termination and pre-mRNA 3′ end processing lead to sensitivity to multiple forms of genome insults and DDR activation^66–70^. In many genes that have multiple pA sites, DDR-mediated inhibition of CPF and CF leads to changes in usage of these alternative pA sites, resulting in preference for longer transcripts^69^. The HU-mediated inhibition of transcription termination (Fig. 4c) is associated with altered pre-mRNA 3′ processing, resulting in reduced cleavage efficiency at the pA sites (Fig. 5), shortening of poly(A) tails (Fig. 8), and mRNAs destabilization (Fig. 7). Since pre-mRNA polyadenylation is dependent on the CPF and CF complexes^62–65,71^, our results are consistent with a model in which HU affects recruitment and/or activity of the CPF and CF complexes, resulting in defects in transcription termination, pre-mRNA cleavage at the pA sites, pre-mRNA polyadenylation, and destabilization of mRNAs. These results are consistent with our observation that deadenylation of mRNAs by Pan2p-Pan3p and Ccr4p-Pop2p-Not deadenylases is not required for the destabilizing effect of HU (Fig. 7).

The reduced efficiency of transcription termination (Fig. 4c) and pre-mRNA cleavage at the pA sites (Fig. 5) in *mec1*∆*sml1*∆ cells indicates that Mec1p has a role in transcriptional termination and pre-mRNA processing. It would be tempting to speculate that at least one of the subunits of CPF, CF or Rat1p is regulated by Mec1p phosphorylation. This is an appealing hypothesis, since Mec1p activation during normal unperturbed S phase or during replication stress would contribute to efficient transcriptional termination, limiting association of RNAPII with chromatin and possible replication-transcription conflicts. The possible role of Mec1p in regulation of CPF, CF or Rat1p is consistent with the reduced cleavage efficiency at the pA sites in *mec1*∆*sml1*∆ cells (Fig. 5). Interestingly, the effect of HU on the cleavage efficiency at the pA sites was observed also in *mec1*∆*sml1*∆ cells, indicating that HU and Mec1p affect pre-mRNA cleavage at the pA sites by independent mechanisms.

The HU-mediated shortening of poly(A) tails appears to require Mec1p (Fig. 8). In contrast, with the exception of *PYK1*, HU destabilizes mRNAs independently of Mec1p (Fig. 7a). Since pre-mRNA polyadenylation and the resulting mRNA stability are dependent on the CPF and CF complexes^62–65,71^, our results are consistent with a model in which HU affects recruitment and/or activity of the CPF and CF complexes to pre-mRNAs, resulting in defects in pre-mRNAs polyadenylation and destabilization of mRNAs. Perhaps the simplest explanation of our results is that Mec1p mediates the effect of HU on recruitment and/or activity of CPF and CF complexes in an mRNA-specific manner.

Overall, our data show that HU globally inhibits RNA synthesis by RNAPI, RNAPII, and RNAPIII. The HU-mediated inhibition of transcription termination is associated with altered pre-mRNA 3′ processing, resulting in reduced cleavage efficiency at the pA sites, shortening of poly(A) tails, and mRNAs destabilization. Our results also indicate that Mec1p-mediated checkpoint signaling is required for normal transcription termination and pre-mRNA processing.

## Experimental procedures

### Yeast strains and media

All yeast strains are listed in Supplementary Table 1. Standard genetic techniques were used to manipulate yeast strains and to introduce mutations from non-W303 strains into the W303 background^72^. Cells were grown in YPD medium (1% yeast extract, 2% Bacto peptone, 2% glucose) or YPR medium (1% yeast extract, 2% Bacto peptone, 2% raffinose). Expression of *GAL1-YLR454* construct was induced by adding 2% galactose for 2 h to cells grown in YPR medium.

### Cell synchronization and flow cytometry

Cell cycle arrest in G1 phase by α-factor and release into S phase was carried out as described^25,37,73,74^ by adding α-factor to 10 µg/ml to cells exponentially growing in YPD medium. Following α-factor addition, the cultures were incubated for 2 h, and an additional 5 µg/ml of α-factor was added and incubation continued for another 1 h. The culture was divided in 2 aliquots, and one aliquot treated for 30 min with HU for the last 30 min of the α-factor arrest. The HU treatment of S-phase cells was performed by arresting WT (W303-1a) cells with α-factor, washing them 2 times with YPD medium pre-warmed to 30 °C, and releasing them into YPD medium or YPD medium containing HU for 30 min. The cell cycle arrest in G1 phase and release into S phase was monitored by flow cytometry of Sytox Green stained cells using Sigma Millipore Guava easy Cyte flow cytometer as described^74^. This protocol yields about 90% of cells arrested in G1 phase and released into S phase^74^.

### 4tU labeling and purification of nascent RNA

*S. cerevisiae* and *S. pombe* nascent RNA was labeled with 4-thiouracil (4tU) as previously described^75,76^. Briefly, *S. cerevisiae* cultures were grown in 30 ml of YPD medium at 30 °C to log phase (A_600nm_ ~ 0.8). In parallel, wild type *S. pombe* cells were grown in 30 ml of YES medium at 32 °C, also to log phase (A_600nm_ ~ 0.8). Newly synthesized RNA in both *S. cerevisiae* and *S. pombe* was labeled for 6 min with 5 mM 4tU. For treated samples, 200 mM HU was added to *S. cerevisiae* 30 min before labeling. After labeling, cells were pelleted and stored at -70 °C. *S. cerevisiae* and *S. pombe* cells were mixed in 3:1 ratio and RNA was extracted and purified using RNeasy kit (74106, Qiagen). None of the solutions used for extraction and purification of RNA contained dithiothreitol or 2-mercaptoethanol. After isolation, 200 µg of the 4tU-labeled RNA dissolved in 100 µl of DEPC-treated RNase free water were incubated at 60 °C for 10 min and cooled down on ice for 2 min. Subsequently, 600 µl of DEPC-treated RNase free water were added, followed by 100 µL of biotinylation buffer (100 mM Tris- HCl pH 7.5, 10 mM EDTA) and 200 µL of 1 mg/mL EZ-link HPDP Biotin (A35390, ThermoFisher). The mixture was incubated for 20 min at 65 °C at 300 rpm protected from light in Eppendorf Thermomixer C. The RNA was phenol–chloroform extracted to remove unincorporated biotin and isopropanol precipitated. The RNA was re-suspended in 100 µL DEPC-treated RNase-free water. Meanwhile, 50 µl of μMACS streptavidin microbeads (130-074-101, Miltenyi Biotec) were equilibrated with 500 µl washing buffer (100 mM Tris–HCl at pH 7.5, 10 mM EDTA, 1 M NaCl, 0.1% Tween20) containing 20 µg of glycogen for 30 min with gentle shaking. Beads were applied to a μcolumn placed in a magnetic stand. After the liquid has drained from the column, the column was removed from the stand and the beads were eluted with 100 µl of the washing buffer. Biotinylated RNA was denatured at 65 °C for 10 min, cooled down for 5 min on ice and incubated with 100 µL of the equilibrated μMACS streptavidin microbeads for 90 min at room temperature with gentle shaking. The microbeads were pipetted to the columns in the magnetic stand, the flow through was reapplied, and the microbeads were washed five times with increasing volumes of washing buffer (600, 700, 800, 900, and 1000 µL). Ultimately, labeled RNA was eluted twice with 200 µL of 0.1 M dithiothreitol and precipitated overnight in 1/10 volume of 3 M NaOAc, 3 volumes of 100% ice cold ethanol and 20 μg of glycogen. The nascent RNA was recovered by centrifugation and resuspended in 60 µL of DEPC-treated RNase-free water.

### Slot blot analysis of biotinylated RNA

*S. cerevisiae* cells were grown, labeled with 4tU, and the RNA isolated and purified as described above. For treated samples, 200 mM HU was added to the cultures 30 min before labeling. RNA was biotinylated using iodoacetyl-biotin (21,333, ThermoFisher Scientific), phenol–chloroform extracted, and purified with RNeasy kit (74106, Qiagen). Slot blot assay was performed as described^77^. Zeta membrane (162-0153, BioRad) was incubated in nuclease-free water for 10 min and assembled in Bio-Dot SF apparatus (162-0161, BioRad). RNA samples were prepared using ice-cold binding buffer (10 mM NaOH, 1 mM EDTA) and applied to the membrane. Wells were rinsed with cold binding buffer and excess buffer was removed by vacuum. RNA was crosslinked to the membrane with UV light at 120 mJoule/cm^2^ for 45 s. The membrane was washed in blocking buffer (0.5 X PBS, 10% SDS, 1 mM EDTA) for 30 min, incubated with 1:1000 streptavidin–horseradish peroxidase (HRP; SA-5004–1; Vector Laboratories) for 15 min and washed six times with PBS containing decreasing concentrations of SDS (10%, 1% and 0.1%, twice each). Membrane-bound HRP was visualized using enhanced chemiluminiscence.

### Real-time RT-qPCR

The procedures to extract total RNA from yeast cells and perform RT-qPCR were as previously described^73,78^. The primers used for RT-qPCR are listed in Supplementary Table 2.

### mRNA decay rates

The half-lives (t_1/2_) of mRNAs were determined using transcriptional shut off with thiolutin as described^37,79,80^. Yeast cells were inoculated to an A_600_ = 0.1 and grown in YPD medium to an A_600_ = 0.8. Thiolutin was added to 8 µg/ml and culture samples were removed during 0 – 120 min incubation. Total RNA was isolated as described above and mRNA levels were determined by RT-qPCR using *RDN25* RNA for normalization. The half-lives of individual mRNAs were determined in Microsoft Excel from logarithmic plots of each remaining mRNA at different times after the transcriptional shutoff.

### ChIP assays

In vivo chromatin crosslinking and immunoprecipitation were performed essentially as described^78^. The only modification of this method was that the spheroplasting step was omitted and whole cells were directly disintegrated with glass beads. Immunoprecipitation was performed with the following antibodies: anti-RNAPII Rpb1p monoclonal antibody (8WG16; 664,912, BioLegend), anti-myc monoclonal antibody (9B11; 2276S; Cell Signaling), and anti-HA monoclonal antibody (F-7; sc-7392; Santa Cruz Biotechnology). The primers used for qPCR are listed in Supplementary Table 3.

### RACE-PAT assay

RACE-PAT (rapid amplification of cDNA ends poly(A) test) assay was performed as described^55–58^. Total RNA (0.1 µg) was reverse transcribed with 5 μM pA reverse primer using LunaScript RT supermix kit (NEB) in a total volume of 20 µl at 55 °C for 10 min and 90 °C for 1 min. The resulting cDNAs (1 µl) were amplified by PCR with One*Taq* Hot Start DNA Polymerase (NEB), with 0.2 μM forward gene specific primer (PYK1-5 forward for *PYK1* and PMA1-pA2 forward for *PMA1*) and 0.2 μM pA primer in 25 μl reaction. PCR conditions were 2 min at 94 °C, followed by 30 cycles of 10 s at 94 °C, 30 s at 58 °C, and 30 s at 72 °C, ending with a 5 min final extension at 72 °C. PCR products were analyzed on 2.5% agarose gel.

### Strand-specific reverse transcription

Total RNA (0.4 µg) was reverse transcribed with 0.5 μM gene-specific primer (PYK1-5 reverse primer for *PYK1* and PMA1-pA2 reverse primer for *PMA1*) in a total volume of 20 µl at 55 °C for 10 min and 90 °C for 1 min. The resulting cDNAs (1 µl) were quantified by qPCR with PYK1-5 forward and reverse primers or PMA1-pA2 forward and reverse primers.

### Statistical analysis

The results represent at least three biologically independent experiments. Numerical results are presented as means ± standard deviations (SD). Data were analyzed by using GraphPad Prism 9.5.1.

### Supplementary Information

Supplementary Information.

## Data Availability

All data are contained within the manuscript and its supplementary information.

## References

[1] Sancar A, Lindsey-Boltz LA, Unsal-Kacmaz K, Linn S (2004). Molecular mechanisms of mammalian DNA repair and the DNA damage checkpoints. Annu. Rev. Biochem..

[2] Putnam CD, Jaehnig EJ, Kolodner RD (2009). Perspectives on the DNA damage and replication checkpoint responses in *Saccharomyces cerevisiae*. DNA Repair.

[3] Ciccia A, Elledge SJ (2010). The DNA damage response: Making it safe to play with knives. Mol. Cell.

[4] Kolodner RD, Putnam CD, Myung K (2002). Maintenance of genome stability in *Saccharomyces cerevisiae*. Science.

[5] Putnam CD, Kolodner RD (2017). Pathways and mechanisms that prevent genome instability in *Saccharomyces cerevisiae*. Genetics.

[6] Bell SP, Labib K (2016). Chromosome duplication in *Saccharomyces cerevisiae*. Genetics.

[7] Pardo B, Crabbé L, Pasero P (2017). Signaling pathways of replication stress in yeast. FEMS Yeast Res..

[8] Lagerwerf S, Vrouwe MG, Overmeer RM, Fousteri MI, Mullenders LH (2011). DNA damage response and transcription. DNA Repair.

[9] Giono LE (2016). The RNA response to DNA damage. J. Mol. Biol..

[10] Capozzo I, Iannelli F, Francia S, d'Adda di Fagagna F (2017). Express or repress? The transcriptional dilemma of damaged chromatin. FEBS J..

[11] Gregersen LH, Svejstrup JQ (2018). The cellular response to transcription-blocking DNA damage. Trends Biochem. Sci..

[12] Silva E, Ideker T (2019). Transcriptional responses to DNA damage. DNA Repair.

[13] Charton R, Muguet A, Griesenbeck J, Smerdon MJ, Conconi A (2019). In yeast cells arrested at the early S-phase by hydroxyurea, rRNA gene promoters and chromatin are poised for transcription while rRNA synthesis is compromised. Mutat. Res..

[14] Bhalla P (2019). Yeast PAF1 complex counters the pol III accumulation and replication stress on the tRNA genes. Sci. Rep..

[15] Loll-Krippleber R, Brown GW (2017). P-body proteins regulate transcriptional rewiring to promote DNA replication stress resistance. Nat. Commun..

[16] Hoffman EA, McCulley A, Haarer B, Arnak R, Feng W (2015). Break-seq reveals hydroxyurea-induced chromosome fragility as a result of unscheduled conflict between DNA replication and transcription. Genome Res..

[17] Shanbhag NM, Rafalska-Metcalf IU, Balane-Bolivar C, Janicki SM, Greenberg RA (2010). ATM-dependent chromatin changes silence transcription in cis to DNA double-strand breaks. Cell.

[18] Charlet-Berguerand N (2006). RNA polymerase II bypass of oxidative DNA damage is regulated by transcription elongation factors. EMBO J..

[19] Tufegdžić Vidaković A (2020). Regulation of the RNAPII pool is integral to the DNA damage response. Cell.

[20] Nguyen VC (2010). Replication stress checkpoint signaling controls tRNA gene transcription. Nat. Struct. Mol. Biol..

[21] Poli J (2016). Mec 1, INO80, and the PAF1 complex cooperate to limit transcription replication conflicts through RNAPII removal during replication stress. Genes Dev..

[22] Hurst V (2021). A regulatory phosphorylation site on Mec1 controls chromatin occupancy of RNA polymerases during replication stress. EMBO J..

[23] Sidorova JM, Breeden LL (1997). Rad53-dependent phosphorylation of Swi6 and down-regulation of CLN1 and CLN2 transcription occur in response to DNA damage in *Saccharomyces cerevisiae*. Genes Dev..

[24] Sidorova JM, Breeden LL (2003). Rad53 checkpoint kinase phosphorylation site preference identified in the Swi6 protein of *Saccharomyces cerevisiae*. Mol. Cell Biol..

[25] Travesa A (2012). DNA replication stress differentially regulates G1/S genes via Rad53-dependent inactivation of Nrm1. EMBO J..

[26] Bastos de Oliveira FM, Harris MR, Brazauskas P, de Bruin RA, Smolka MB (2012). Linking DNA replication checkpoint to MBF cell-cycle transcription reveals a distinct class of G1/S genes. EMBO J..

[27] Jaehnig EJ, Kuo D, Hombauer H, Ideker TG, Kolodner RD (2013). Checkpoint kinases regulate a global network of transcription factors in response to DNA damage. Cell Rep..

[28] Zhou C (2016). Profiling DNA damage-induced phosphorylation in budding yeast reveals diverse signaling networks. Proc. Natl. Acad. Sci. U. S. A..

[29] Lavigne MD, Konstantopoulos D, Ntakou-Zamplara KZ, Liakos A, Fousteri M (2017). Global unleashing of transcription elongation waves in response to genotoxic stress restricts somatic mutation rate. Nat. Commun..

[30] Williamson L (2017). UV irradiation induces a non-coding RNA that functionally opposes the protein encoded by the same gene. Cell.

[31] Gyenis A (2014). UVB induces a genome-wide acting negative regulatory mechanism that operates at the level of transcription initiation in human cells. PLoS Genet..

[32] Rockx DA (2000). UV-induced inhibition of transcription involves repression of transcription initiation and phosphorylation of RNA polymerase II. Proc. Natl. Acad. Sci. U. S. A..

[33] Galli A, Schiestl RH (1996). Hydroxyurea induces recombination in dividing but not in G1 or G2 cell cycle arrested yeast cells. Mutat. Res..

[34] Szikriszt B (2016). A comprehensive survey of the mutagenic impact of common cancer cytotoxics. Genome Biol..

[35] Zhao X, Chabes A, Domkin V, Thelander L, Rothstein R (2001). The ribonucleotide reductase inhibitor Sml1 is a new target of the Mec1/Rad53 kinase cascade during growth and in response to DNA damage. EMBO J..

[36] Pelechano V, Chávez S, Pérez-Ortín JE (2010). A complete set of nascent transcription rates for yeast genes. PLoS ONE.

[37] Bhagwat M (2021). Replication stress inhibits synthesis of histone mRNAs in yeast by removing Spt10p and Spt21p from the histone promoters. J. Biol. Chem..

[38] Kaplan CD, Laprade L, Winston F (2003). Transcription elongation factors repress transcription initiation from cryptic sites. Science.

[39] Keogh MC (2005). Cotranscriptional set2 methylation of histone H3 lysine 36 recruits a repressive Rpd3 complex. Cell.

[40] Carrozza MJ (2005). Histone H3 methylation by Set2 directs deacetylation of coding regions by Rpd3S to suppress spurious intragenic transcription. Cell.

[41] Joshi AA, Struhl K (2005). Eaf3 chromodomain interaction with methylated H3–K36 links histone deacetylation to Pol II elongation. Mol. Cell.

[42] Cheung V (2008). Chromatin- and transcription-related factors repress transcription from within coding regions throughout the *Saccharomyces cerevisiae* genome. PLoS Biol..

[43] Kim M, Ahn SH, Krogan NJ, Greenblatt JF, Buratowski S (2004). Transitions in RNA polymerase II elongation complexes at the 3' ends of genes. EMBO J..

[44] Kim M (2004). The yeast Rat1 exonuclease promotes transcription termination by RNA polymerase II. Nature.

[45] Schwabish MA, Struhl K (2006). Asf1 mediates histone eviction and deposition during elongation by RNA polymerase II. Mol. Cell.

[46] Kamieniarz-Gdula K, Proudfoot NJ (2019). Transcriptional control by premature termination: A forgotten mechanism. Trends Genet..

[47] Luo W, Johnson AW, Bentley DL (2006). The role of Rat1 in coupling mRNA 3'-end processing to transcription termination: Implications for a unified allosteric-torpedo model. Genes Dev..

[48] Lykke-Andersen S, Jensen TH (2007). Overlapping pathways dictate termination of RNA polymerase II transcription. Biochimie.

[49] Mischo HE, Proudfoot NJ (2013). Disengaging polymerase: Terminating RNA polymerase II transcription in budding yeast. Biochim. Biophys. Acta..

[50] Baejen C (2017). Genome-wide analysis of RNA polymerase II termination at protein-coding genes. Mol. Cell.

[51] Mason PB, Struhl K (2005). Distinction and relationship between elongation rate and processivity of RNA polymerase II in vivo. Mol. Cell.

[52] Parker R (2012). RNA degradation in *Saccharomyces cerevisiae*. Genetics.

[53] Tharun S, Parker R (2001). Targeting an mRNA for decapping: Displacement of translation factors and association of the Lsm1p-7p complex on deadenylated yeast mRNAs. Mol. Cell.

[54] Chowdhury A, Mukhopadhyay J, Tharun S (2007). The decapping activator Lsm1p-7p-Pat1p complex has the intrinsic ability to distinguish between oligoadenylated and polyadenylated RNAs. RNA.

[55] Sallés FJ, Darrow AL, O'Connell ML, Strickland S (1992). Isolation of novel murine maternal mRNAs regulated by cytoplasmic polyadenylation. Genes Dev..

[56] Sallés FJ, Strickland S (1995). Rapid and sensitive analysis of mRNA polyadenylation states by PCR. PCR Methods Appl..

[57] Sallés FJ, Richards WG, Strickland S (1999). Assaying the polyadenylation state of mRNAs. Methods.

[58] Brouze A, Krawczyk PS, Dziembowski A, Mroczek S (2022). Measuring the tail: Methods for poly(A) tail profiling. Wiley Interdiscip. Rev. RNA.

[59] Tudek A, Lloret-Llinares M, Jensen TH (2018). The multitasking polyA tail: Nuclear RNA maturation, degradation and export. Philos. Trans. R. Soc. Lond. B. Biol. Sci..

[60] Luo W, Bentley D (2004). A ribonucleolytic rat torpedoes RNA polymerase II. Cell.

[61] Hyman LE, Moore CL (1993). Termination and pausing of RNA polymerase II downstream of yeast polyadenylation sites. Mol. Cell. Biol..

[62] Casañal A (2017). Architecture of eukaryotic mRNA 3'-end processing machinery. Science.

[63] Kühn U (2009). Poly(A) tail length is controlled by the nuclear poly(A)-binding protein regulating the interaction between poly(A) polymerase and the cleavage and polyadenylation specificity factor. J. Biol. Chem..

[64] Stewart M (2019). Polyadenylation and nuclear export of mRNAs. J. Biol. Chem..

[65] Turtola M (2021). Three-layered control of mRNA poly(A) tail synthesis in *Saccharomyces cerevisiae*. Genes Dev..

[66] Gaillard H, Aguilera A (2014). Cleavage factor I links transcription termination to DNA damage response and genome integrity maintenance in *Saccharomyces cerevisiae*. PLoS Genet..

[67] Kuehner JN, Kaufman JW, Moore C (2017). Stimulation of RNA Polymerase II ubiquitination and degradation by yeast mRNA 3'-end processing factors is a conserved DNA damage response in eukaryotes. DNA Repair.

[68] Dutertre M, Sfaxi R, Vagner S (2021). Reciprocal links between pre-messenger RNA 3'-end processing and genome stability. Trends Biochem. Sci..

[69] Graber JH (2013). DNA damage induces targeted, genome-wide variation of poly(A) sites in budding yeast. Genome Res..

[70] Srividya I, Tirupataiah S, Mishra K (2012). Yeast transcription termination factor Rtt103 functions in DNA damage response. PLoS ONE.

[71] Mandart E, Parker R (1995). Effects of mutations in the *Saccharomyces cerevisiae RNA14*, *RNA15*, and *PAP1* genes on polyadenylation in vivo. Mol. Cell. Biol..

[72] Sherman F (1991). Getting started with yeast. Methods Enzymol..

[73] Bu P (2019). DNA damage response activates respiration and thereby enlarges dNTP pools to promote cell survival in budding yeast. J. Biol. Chem..

[74] Nagar S, Mehta R, Kaur P, Liliah RT, Vancura A (2023). Tolerance to replication stress requires Dun1p kinase and activation of the electron transport chain. Biochim. Biophys. Acta Mol. Cell Res..

[75] Baptista T (2018). SAGA is a general cofactor for RNA polymerase II transcription. Mol. Cell.

[76] Baptista T, Devys D (2018). *Saccharomyces cerevisiae* metabolic labeling with 4-thiouracil and the quantification of newly synthesized mRNA as a proxy for RNA polymerase II activity. J. Vis. Exp..

[77] Rädle B (2013). Metabolic labeling of newly transcribed RNA for high resolution gene expression profiling of RNA synthesis, processing and decay in cell culture. J. Vis. Exp..

[78] Galdieri L, Vancura A (2012). Acetyl-CoA carboxylase regulates global histone acetylation. J. Biol. Chem..

[79] Coller J (2008). Methods to determine mRNA half-life in *Saccharomyces cerevisiae*. Methods Enzymol..

[80] Passos DO, Parker R (2008). Analysis of cytoplasmic mRNA decay in *Saccharomyces cerevisiae*. Methods Enzymol..

