# Supplementary material for: Hydroxyurea and inactivation of checkpoint kinase MEC1 inhibit transcription termination and pre-mRNA cleavage at polyadenylation sites in budding yeast
Source: Sci Rep. 2023 Aug 11;13:13106. doi: 10.1038/s41598-023-40294-3 (PMC10421882; doi:10.1038/s41598-023-40294-3)

Supplementary Information

Hydroxyurea and inactivation of checkpoint kinase *MEC1* inhibit transcription termination and pre-mRNA cleavage at the polyadenylation sites in budding yeast

Pritpal Kaur[1], Shreya Nagar[1], Riddhi Mehta, Kyle Sahadeo, and Ales Vancura[*]

Department of Biological Sciences, St. John's University, Queens, New York, USA

[1]These authors contributed equally

Supplementary Table 1

Yeast strains used in this study

Strain	Genotype	Source/Ref.
W303-1a	*MATa ade2-1 his3-11,15 leu2-3,112 trp1-1 ura3-1 ssd1-d2 can1-100*	R. Rothstein
W303-1α	*MATα ade2-1 his3-11,15 leu2-3,112 trp1-1 ura3-1 ssd1-d2 can1-100*	R. Rothstein
W303	*MATa/MATα ade2-1/ade2-1 his3-11,15/his3-11,15 leu2-3,112/leu2-3,112 trp1-1/trp1-1ura3-1/ura3-1 can1-100/can1-100*	R. Rothstein
SN141	*MATa ade2-1 his3-11,15 leu2-3,112 trp1-1 ura3-1 ssd1-d2 can1-100 dun1::HIS3*	This study
SN159	*MATa ade2-1 his3-11,15 leu2-3,112 trp1-1 ura3-1 ssd1-d2 can1-100 tel1::HIS3*	1
SN136	*MATα ade2-1 his3-11,15 leu2-3,112 trp1-1 ura3-1 ssd1-d2 can1-100 chk1::HIS3*	1
SN117	*MATa ade2-1 his3-11,15 leu2-3,112 trp1-1 ura3-1 ssd1-d2 can1-100 mec1::HIS3 sml1::KAN*	1
LG606	*MATa ade2-1 his3-11,15 leu2-3,112 trp1-1 ura3-1 ssd1-d2 can1-100 rad53::KAN sml1::HYG*	1
AD066	*MATα ade2-1 his3-11,15 leu2-3,112 trp1-1 ura3-1 ssd1-d2 can1-100 SPT15-3HA::URA3*	2
MB163	*MATa ade2-1 his3-11,15 leu2-3,112 trp1-1 ura3-1 ssd1-d2 can1-100 mec1::HIS3 sml1::KAN SPT15-3HA::URA3*	3
MB123	*MATa ade2-1 his3-11,15 leu2-3,112 trp1-1 ura3-1 ssd1-d2 can1-100 pan2::URA3*	3
SM096	*MATa ade2-1 his3-11,15 leu2-3,112 trp1-1 ura3-1 ssd1-d2 can1-100 ccr4::URA3*	3
MB129	*MATa ade2-1 his3-11,15 leu2-3,112 trp1-1 ura3-1 ssd1-d2 can1-100 dcp2::HIS3*	This study
MB115	*MATa ade2-1 his3-11,15 leu2-3,112 trp1-1 ura3-1 ssd1-d2 can1-100 xrn1::URA3*	3
MB153	*MATa ade2-1 his3-11,15 leu2-3,112 trp1-1 ura3-1 ssd1-d2 can1-100 spt21::HIS3*	This study
MZ672	*MATa ade2-1 his3-11,15 leu2-3,112 trp1-1 ura3-1 ssd1-d2 can1-100 spt10::KAN*	4
SJ027	*MATa ade2-1 his3-11,15 leu2-3,112 trp1-1 ura3-1 ssd1-d2 can1-100 rad9:: KAN*	This study
SJ015	*MATa ade2-1 his3-11,15 leu2-3,112 trp1-1 ura3-1 ssd1-d2 can1-100 mrc1:: KAN*	This study
MZ576	*MATa ade2-1 his3-11,15 leu2-3,112 trp1-1 ura3-1 ssd1-d2 can1-100 asf1::HIS3*	5

MZ642	*MATa ade2-1 his3-11,15 leu2-3,112 trp1-1 ura3-1 ssd1-d2 can1-100 rtt106::KAN*	5
MZ533	*MATa ade2-1 his3-11,15 leu2-3,112 trp1-1 ura3-1 ssd1-d2 can1-100 cac1::LEU2*	5
MZ700	*MATa ade2-1 his3-11,15 leu2-3,112 trp1-1 ura3-1 ssd1-d2 can1-100 hir1::HIS3*	5
CMY125	*MATa his3-Δ1 leu2-Δ0 met15-Δ0 ura3-Δ0* P_{GAL1}*-YLR454w*	6
SN691	*MATa ade2-1 his3-11,15 leu2-3,112 trp1-1 ura3-1 ssd1-d2 can1-100* P_{GAL1}*-YLR454w*	This study
SN684	*MATa ade2-1 his3-11,15 leu2-3,112 trp1-1 ura3-1 ssd1-d2 can1-100 mec1::HIS3 sml1::KAN* P_{GAL1}*-YLR454w*	This study
YSB1796	*MATa ura3-52,leu2Δ1, trp1Δ63,his3ΔLEU2/KanR, rat1-1-TAP::HIS3*	7
JS311-A190MN	*MATα his3Δ200,leu2Δ1,met*15Δ0, *trp1Δ63,ura3-167, RDN1::Ty1-MET15,mURA3/HIS3; RPA190-MNase-3X-HA::KanMX6*	8
RET1-13MYC	*MAT*α *his*3Δ1 *leu*2Δ0 *lys*2Δ0 *ura*3Δ0 *RET1-13MYC-KanMX*	9
DBY548	*MATa ade2-1 his3-11,15 leu2-3,112 trp1-1 ura3-1 GAL1-ADH4::TRP1*	10
RM168	*MATa ade2-1 his3-11,15 leu2-3,112 trp1-1 ura3-1 mec1::HIS3 sml1::KAN GAL1-ADH4::TRP1*	This study

References

1. Bu, P., Nagar, S., Bhagwat, M., Kaur, P., Shah, A., Zeng, J., Vancurova, I., and Vancura, A. (2019) DNA damage response activates respiration and thereby enlarges dNTP pools to promote cell survival in budding yeast. *J. Biol. Chem.* **294**, 9771-9786
2. Demczuk, A., Guha, N., Nguyen, P.H., Desai, P., Chang, J., Guzinska, K., Rollins, J., Ghosh, C.C., Goodwin, L., and Vancura. A. (2008) *Saccharomyces cerevisiae* phospholipase C regulates transcription of Msn2p-dependent stress-responsive genes. *Eukaryot. Cell.* **7**, 967-979
3. Bhagwat, M., Nagar, S., Kaur, P., Mehta, R., Vancurova, I., and Vancura, A. (2021) Replication stress inhibits synthesis of histone mRNAs in yeast by removing Spt10p and Spt21p from the histone promoters. *J. Biol. Chem.* **297**, 101246
4. Mehrotra, S., Galdieri, L., Zhang, T., Zhang, M., Pemberton, L. F., and Vancura, A. (2014) Histone hypoacetylation-activated genes are repressed by acetyl-CoA- and chromatin-mediated mechanism. *Biochim. Biophys. Acta.* **1839**, 751–763
5. Galdieri, L., Zhang, T., Rogerson, D., and Vancura, A. (2016) Reduced Histone Expression or a Defect in Chromatin Assembly Induces Respiration. *Mol. Cell. Biol.* **36**, 1064-1077
6. Wong, C. M., Qiu, H., Hu, C., Dong, J., and Hinnebusch, A. G. (2007). Yeast cap binding complex impedes recruitment of cleavage factor IA to weak termination sites. *Mol. Cell. Biol.*, **27**, 6520–6531
7. Kim, M., Krogan, N. J., Vasiljeva, L., Rando, O. J., Nedea, E., Greenblatt, J. F., and Buratowski, S. (2004) The yeast Rat1 exonuclease promotes transcription termination by RNA polymerase II. *Nature*, **432**, 517–522

8. Charton, R., Muguet, A., Griesenbeck, J., Smerdon, M.J., and Conconi, A. (2019) In yeast cells arrested at the early S-phase by hydroxyurea, rRNA gene promoters and chromatin are poised for transcription while rRNA synthesis is compromised. *Mutat. Res.* **815**, 20–29
9. Soragni, E., and Kassavetis, G. A. (2008). Absolute gene occupancies by RNA polymerase III, TFIIIB, and TFIIIC in Saccharomyces cerevisiae. *J. Biol. Chem.* **283**, 26568–26576
10. Luo, W., Johnson, A.W., and Bentley, D.L. (2006). The role of Rat1 in coupling mRNA 3'-end processing to transcription termination: implications for a unified allosteric-torpedo model. *Genes Dev.* **20**, 954-965

Supplementary Table 2

Primers for RT-qPCR

Primer Name	Forward (5'-3')	Reverse (5'-3')
**SNR52**	TGATGAATGACATTAGCGTGAACA	GAAGGAAGGCAACATAAGTTTTTCT
**RPR1**	CCACCTATGGGCGGGTTATC	AGGCCGAACTCCGTGAATTT
**SUP4**	TCGGTAGCCAAGTTGGTTTAAGG	TCTCCCGGGGGCGAGTC
**SPTUB**	CCGCTGGTGGAAAGTATGTT	GCCAATTCAGCACCTTCAGT
**SCR1**	CCTTTGGGCAAGGGATAGTT	TTTACGACGGAGGAAAGACG
**SNR6**	CGAAGTAACCCTTCGTGGAC	TCATCCTTATGCAGGGGAAC
**YEF3**	ATCTGTTGCCACTGCTGACA	TAGCAGCGGTCTTCTTGTCC
**RPL3**	AGAGAGCTGCCTCCATCAGA	CAGCTTCGACAACTTCACGC
**PYK1**	TTGTTGCTGGTTCTGACTTGAG	CAATGTTCAAACCAGCCTTTCTC
**25S**	GGAATGTAGCTTGCCTCGGT	TTACGTCGCAGTCCTCAGTC
**5S**	ACCAGAAAGCACCGTTTCCC	GCACCTGAGTTTCGCGTATG
**FLO8-5' region**	CCCACGGAACAACCGTACAT	TCCGAATTTTCCGCCGTAGG
**FLO8-3' region**	TGTGGGTACAACTTTGGGTCC	ATCCGGTCCTTGGTCTTCAA
**VPS72-5' region**	TCTAGGAGGTCTAATGCCGGT	TCTTCCTGAAACAACAGCCCT
**VPS72-3' region**	CCAAGGACTGGTGTGCCTTA	TACCGCCGTTTTTGAACCCA
**PMA1-2**	GTGTCGACGACGAAGACAGT	ACCGTAAGATGGGTCAGTTTGT
**PMA1-6**	AAGTGTCGAAGACTTCATGGCT	AATGCTACTTCAACAGGATTAGGT
**ADH1-2**	ACGAATCCCACGGTAAGTTGG	CAGTGTGACAGACACCAGAGT
**ADH1-4**	CGTCGGTAACAGAGCTGACA	GCAAGGTAGACAAGCCGACA
**PYK1-2**	ACGTTGTTGCTGGTTCTGAC	CCAGCCTTTCTCAAAGCAACC
**PYK1-4**	AGCCCGTATCAACTTCGGTA	TGTTGGAGTGACCAGCACC
**RPL3-2**	AGTACGAAGCACCACGTCAC	AGCAACTGGCTTGGATCTGT
**RPL3-5**	TGGTTTCGTCCACTACGGTG	AGCTGACTTCTTCCAAAGCCT
**YEF3-2**	ATCTGTTGCCACTGCTGACA	TAGCAGCGGTCTTCTTGTCC
**YEF3-7**	CCGGTACATGGCAAAGACCT	TTGACGGCCCAGACTTCTTC
**PMA1-pA1**	CACGAAAAGGAAACCTAATCCTGT	GCGGCTTATTCTTGTTGGCT
**PMA1-pA2**	CAACACGCACATTTCAACTTTAAT	TCTTTGTCCCTCTATACAAACATCT
**pA**		GCGAGCTCCGCGGCCGCGTTTTTTTTTTTT

Supplementary Table 3

Primers for CHIP

Primer name	Forward (5'-3')	Reverse (5'-3')
**PMA1-1**	CCAATTATGACCGGTGACGAAA	ATCGAAACTAATGGAGGGGAGC
**PMA1-2**	GTGTCGACGACGAAGACAGT	ACCGTAAGATGGGTCAGTTTGT
**PMA1-3**	ACGAAGTCGTCCCAGGTGA	TGGTCACCGTAATGTTTGTCG
**PMA1-4**	CCAGCTGTCGTTACCACCAC	GATTTCGACACCAGCCAAGG
**PMA1-5**	ATGCTTTGAAGACCTCCAGAC	AGCAATCCATAGACCCAAGAAGA
**PMA1-6**	AAGTGTCGAAGACTTCATGGCT	AATGCTACTTCAACAGGATTAGGT
**PMA1-7**	AGCCAACAAGAATAAGCCGC	AAATTTTAAGGTGTGTGTGTGGA
**PMA1-8**	TGCAAGATGTTTGTATAGAGGGACA	AGAAGAGCTGGGCAGGAACT
**PMA1-9**	CTCGCTTACGTTTATGCGCC	GGAGGCGGCCTTCAATCAT
**ADH1-1**	ACAGCACCAACAGATGTCGT	AAGGCCGTATACCGTTGCTC
**ADH1-2**	ACGAATCCCACGGTAAGTTGG	CAGTGTGACAGACACCAGAGT
**ADH1-3**	GTCTGCTAACTTGATGGCCG	TCACCACCGTCAATACCCAA
**ADH1-4**	CGTCGGTAACAGAGCTGACA	GCAAGGTAGACAAGCCGACA
**ADH1-5**	GGTCAGGTTGCTTTCTCAGG	GGGTGAAATGGGGAGCGATT
**PYK1-1**	TCCTTTCCTTCCCATATGATGCT	GGTTCTTGGAAATGAAAAGTTACCA
**PYK1-2**	ACGTTGTTGCTGGTTCTGAC	CCAGCCTTTCTCAAAGCAACC
**PYK1-3**	TTCATCAGAACCGCCAACGA	TGGCAACCATAACACCGTCA
**PYK1-4**	AGCCCGTATCAACTTCGGTA	TGTTGGAGTGACCAGCACC
**PYK1-5**	AGACATGGTTTTTCTTTTCAACTCA	CCAAAAATGCAACACCTCATCG
**PYK1-6**	GGATGGCGAAAGGATACGCT	AACGAAGGCCAGAAGCTGAA
**YEF3-1**	CCCACCCATGCATAACCCTA	AGATAATTATACTCGAGGAAGCGAA
**YEF3-2**	ATCTGTTGCCACTGCTGACA	TAGCAGCGGTCTTCTTGTCC
**YEF3-3**	GGAAGTCAAGGCTGCTGCTA	GGAACTTCAGTTGGGTCAGC
**YEF3-4**	ATTGCTGACCCAGAAGCCAG	AGCAACGGTTTCGTCCTTCA
**YEF3-5**	TGCTTTGTCTGGTGGTTGGA	ACCAAGCAACGTTGACGGTA
**YEF3-6**	GCCGGTATCCACTCCAGAAG	ACGGACATCATTGGAACCCA
**YEF3-7**	CCGGTACATGGCAAAGACCT	TTGACGGCCCAGACTTCTTC
**YEF3-8**	AAAGCGTTCCATTAGTCAGACA	ACCGAAAAGGGTATGAGGCA
**RPL3-1**	ACTCACGCACACTGGAATGA	GAAAACAGTTGTGCGTCGCT
**RPL3-2**	AGTACGAAGCACCACGTCAC	AGCAACTGGCTTGGATCTGT
**RPL3-3**	AAGTACGCTTCCGTCGTCAG	TCGAAATGTTCACGAGCCCA
**RPL3-4**	AAGGGTCACGGTTTCGAAGG	CCACATAACGTGGGCTGGAT

**RPL3-5**	TGGTTTCGTCCACTACGGTG	AGCTGACTTCTTCCAAAGCCT
**RPL3-6**	GACCCGCATGCGATTATGTT	GAGGTCCAAGTCAAAACGGC
**YLR454w-1**	ACCGTCAGGCTAAAATCCGT	AGCCCCCACCATTATCTTCG
**YLR454w-2**	GGCCGTCTCAGGAATACACA	ACTCCTTTAGTTGGCCCCAT
**YLR454w-3**	TTACTCGTTGTTCGTGCCCA	GAACCACCCCAAGTTACTCGT
**YLR454w-4**	CTTTAGCAAGTGGCAAGGGC	TGCTTTGTCTGTTCCTTCTGGT
**YLR454w-5**	TCCAACCAAACTTTGAGGGTCA	TGGTGAAGTGTCGTCAGCAA
**YLR454W-6**	AAACAAGGTCACACGAAAACCA	TGCCTTAGTTATCGTTCAAATGC
**POL1N**	TGGTAGGCTGATATGTGATATCGC	AACGGCTTATGCTCCTTTTCAC
**RDN37-1**	CGGGGCACCTGTCACTTT	TCTTTTGCCCTCTCTGTCGC
**RDN37-2**	GCAGAGAGACCTGAAAAAGCA	CTACTGGCAGGATCAACCAGA
**RDN37-3**	AACCTTGAGTCCTTGTGGCT	ACCAACAAAATAGAACCAAACGT
**RDN37-4**	GGTTTCAAGCCGATGGAAGT	CCAAGGTTAGACTCGCTGGC
**RDN37-5**	TTAAGTGCGCGGTCTTGCTA	CGATTGCTCGAATGCCCAAA
**RDN37-6**	AACGGATCTCTTGGTTCTCG	GTGCGTTCAAAGATTCGATG
**RDN37-7**	GGTTTCTCTGCGTGCTTGAG	ATTGTTCGCCTAGACGCTCTC
**RDN37-8**	GGGCATTTGATCAGACATGGT	GGCAGTATTCCCACAGGCTA
**RDN37-9**	AACAGCTTATCACCCCGGAA	TGCGGTTATCAGTACGACCT
**RDN37-10**	TCTGCTGAGATTAAGCCTTTGT	TTCTCTCTAAACTAGGCCCCG
**RDN37-11**	AAGATGGGTTGAAAGAGAAGGG	TCATATCAAAGGCATGTCCTGT
**SNR52**	TGATGAATGACATTAGCGTGAACA	GAAGGAAGGCAACATAAGTTTTTCT
**RPR1**	CCACCTATGGGCGGGTTATC	AGGCCGAACTCCGTGAATTT
**SUP4**	TCGGTAGCCAAGTTGGTTTAAGG	TCTCCCGGGGGCGAGTC
**SCR1**	CCTTTGGGCAAGGGATAGTT	TTTACGACGGAGGAAAGACG
**SNR6**	CGAAGTAACCCTTCGTGGAC	TCATCCTTATGCAGGGGAAC
**HYP2**	TGAAACTGCTGACGCTGGTT	ACTTTAGCGTGACCGTGCTT
**PGK1**	TGGACTTGAAGGACAAGCGT	GATGGTTGGCAAAGCAGCAA
**ACT1**	TATGTGTAAAGCCGGTTTTGC	GACAATACCGTGTTCAATTGGG
**5S**	ACCAGAAAGCACCGTTTCCC	GCACCTGAGTTTCGCGTATG
**MUP1**	CAACGGTGCCTCCGATTTTG	GAGGAAACGGCGAAAACACC
**PDC1**	ATTGCCGGTTCTTACGCTGA	TCACCGTTACCCAAGGTGTG
**EDC1**	GCGAGGGGAAATGCTGCTAA	GGGAAGAAGGTGGAGAGTGC
**ADH4-1**	CCATCAAGGCTTTGCACGTT	CATGGCGTGTTCAGCCAAAA
**ADH4-2**	CCGAGACCGGTACGTTTGTT	GCATCCAACTGAAACCCACC
**ADH4-3**	ATGTAACCTTACCAGCCGCA	GTCTGATGGGGGTGCTGTAG

**ADH4-4**	TTGCGTTATTCAAGAGCGATGA	ACTACTTTCCACGAATGTACAGAT
**ADH4-5**	AGAAAGTAGCGGACGGTGTC	TTTGGATCACCGAAAGCTGG

Supplementary Fig. 1a

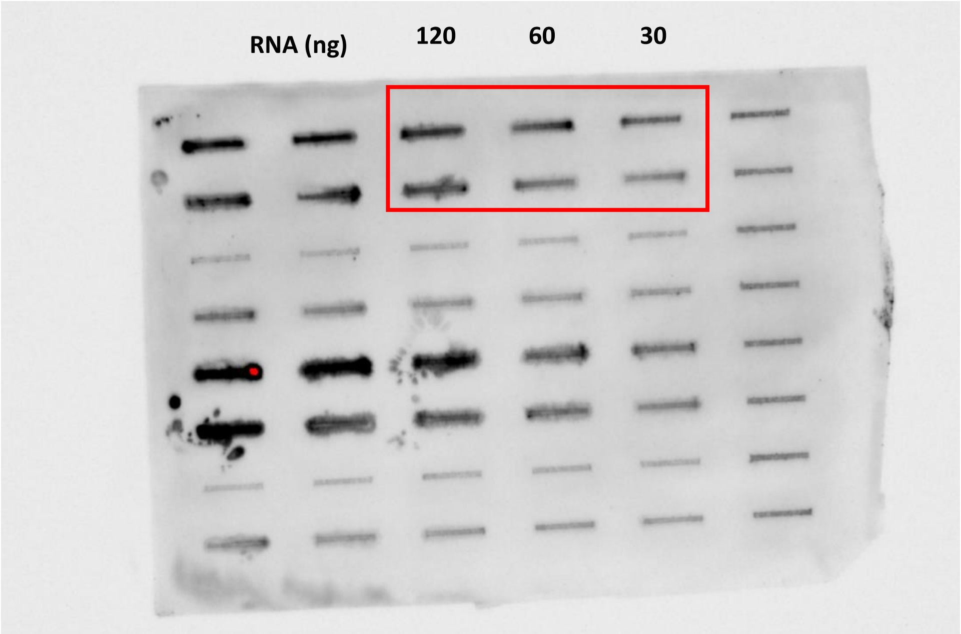

Supplementary Figure 8a

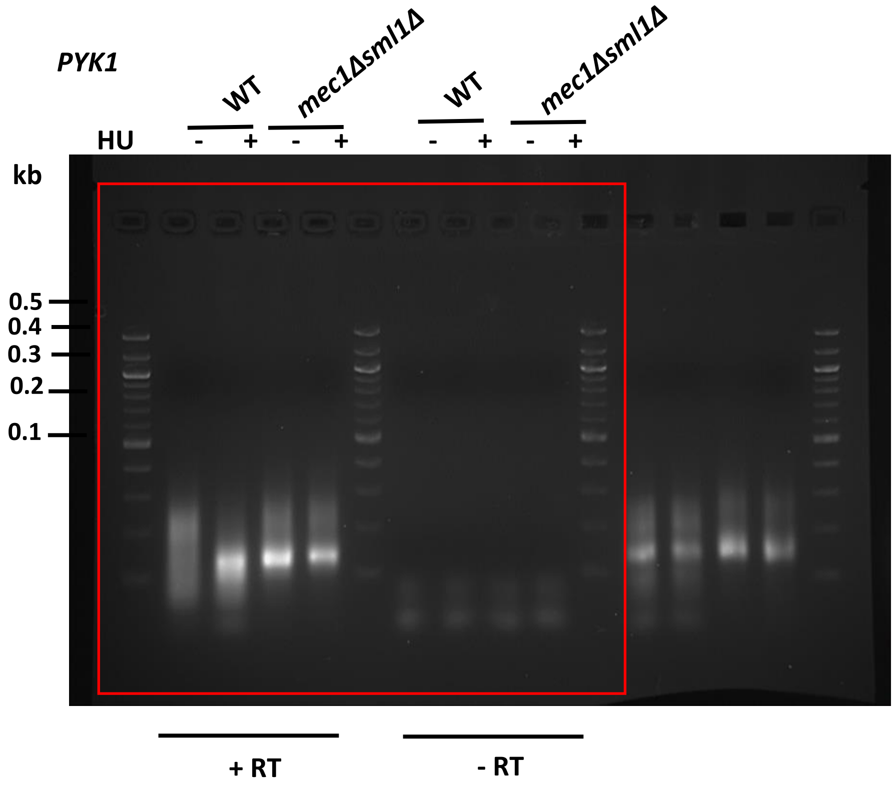

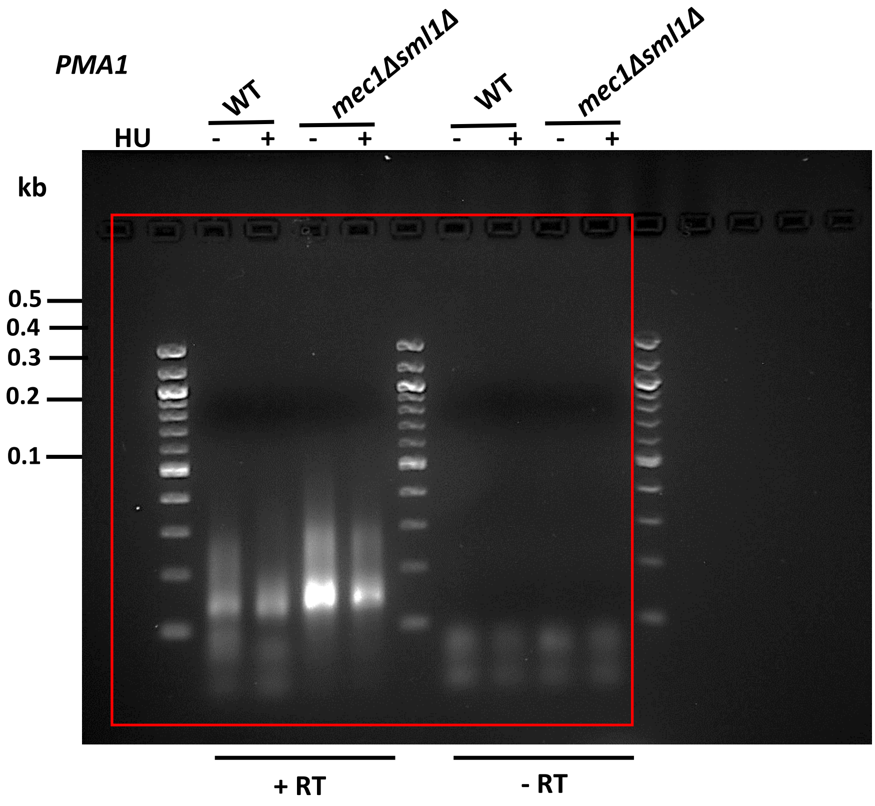

Supplement: Supplementary file 1 — Supplementary Information. [file 41598_2023_40294_MOESM1_ESM.pdf]
